# Comparative Chloroplast Genome and Phylogenetic Analyses of *Anna* and *Lysionotus* (Gesneriaceae) Along the Sino-Vietnamese Border

**DOI:** 10.3390/biology15040352

**Published:** 2026-02-18

**Authors:** Jiahui Li, Zhangping Huang, Weibin Xu, Changhong Guo

**Affiliations:** 1 Key Laboratory of Molecular and Cytogenetics, College of Life Science and Technology, Harbin Normal University, Harbin 150025, China; hsdswlijiahui@163.com; 2 Guangxi Key Laboratory of Plant Conservation and Restoration Ecology in Karst Terrain, Guangxi Institute of Botany, Guangxi Zhuang Autonomous Region and Chinese Academy of Sciences, Guilin 541006, China; 3 State Key Laboratory of Crop Gene Resources and Breeding, Institute of Crop Sciences, Chinese Academy of Agricultural Sciences (CAAS), Beijing 100081, China; huangzhangping@caas.cn; 4 Nanfan Research Institute, Chinese Academy of Agricultural Sciences, Sanya 572024, China

**Keywords:** sympatric species, chloroplast genome, adaptive evolution, phylogenetic relationships, convergent evolutionary

## Abstract

Sympatric species share identical geographical spaces, climatic conditions and survival pressures. Comparative chloroplast genomes among *Anna* and *Lysionotus* sympatric species enable exploration of genome-wide evolutionary dynamics of sympatric species. In this study, we assembled and annotated 10 complete chloroplast genomes, representing sympatric species distributed along the Sino-Vietnamese border. We conducted a comparison of chloroplast genomes, characterized their adaptive evolution and used multiple methods to clarify their phylogenetic relationships. Key findings included the following: 1. The number of CDs, rRNA and tRNA varied among different species, whereas they were relatively conserved between the two genera; 2. *psaB-psaA*, *trnL-UAG* and *ndhD-psaC* were identified as potential molecular markers for *Anna* species, with *clpP* and *ycf1* proposed as effective molecular markers for *Lysionotus* species; 3. the types of simple sequence repeats (SSRs) and large sequence repeats (LRSs) showed a higher conservation in *Lysionotus* compared with *Anna*; 4. the codon usage preferences of the two genera showed convergent evolutionary trends and natural selection played a dominant role, with *ycf1* and *atpH* being confirmed as significantly positively selected genes; 6. phylogenetic analyses using multiple approaches (ML, BI and NJ) consistently verified that *Anna* and *Lysionotus* each formed a well-supported monophyletic group. This study offers molecular insights into adaptation and differentiation patterns among distinct plant genera inhabiting the same extreme habitat.

## Simple Summary

The Sino-Vietnamese border region is a hot spot for *Lysionotus* diversification and the main natural distribution area of its closely related genus, *Anna*. These plants are not only carriers for revealing the adaptive evolution rules of karst habitats, but also key taxa for deciphering the mechanisms of species differentiation and dispersal across heterogeneous habitats. We first reported and compared the complete chloroplast genomes of 10 species from these two genera distributed along the Sino-Vietnamese border, explored chloroplast differences, adaptive signals and phylogenetic relationships, identified candidate molecular markers, analyzed selection patterns and genomic variations, and verified the monophyly of the two genera. These results fill the gap in chloroplast genome data of endemic plants in this region, enrich genomic resources, provide a typical case for the adaptive evolution of endemic plants in Gesneriaceae, and lay a foundation for subsequent research on conservation genetics and molecular evolution of related species.

## 1. Introduction

The border area between China and Vietnam, as one of the global biodiversity hotspots, has attracted much attention due to its unique geographical location and complex ecological environment [1]. This area spans the Tropic of Cancer, with significant temperature fluctuations. The terrain encompasses various types such as high mountains, hills and river valleys, including the eastern section of the Yunnan–Guizhou Plateau and the karst landforms from Guilin [2]. Thus, the complex topographical pattern and diverse climatic conditions jointly create highly heterogeneous habitats, providing ideal conditions for species differentiation and the formation of endemism [3]. There are 5267 species of vascular plants belonging to 1642 genera and 247 families in this area [4]. During the process of adaptive evolution, these endemic plants have developed specific mechanisms to adapt to the barren karst soil, high humidity river valleys, and weak light environment beneath dense forests [5].

Although there has been some research accumulation on plant diversity in the border area between China and Vietnam, the research mainly focuses on species cataloging and descriptions of new species [4]. The exploration of the adaptive evolutionary mechanisms of endemic plants, the interspecific phylogenetic relationships and the mechanisms of endangerment remains insufficient [6]. Carrying out further in-depth research on the unique plant groups in this region not only helps to reveal the ecological and genetic mechanisms behind the formation of species in the tropical–subtropical transition zone, but also provides a scientific basis for formulating cross-border biodiversity conservation strategies [7]. Especially, against the background of current global climate change, deciphering the environmental adaptation strategies of these plants holds important practical value for predicting community dynamics and guiding ecological restoration [8,9].

*Lysionotus* D.Don [10] comprises approximately 30 species of epiphytic or lithophytic shrubs characterized by succulent stems and heterophyllous leaves, with around 15 species distributed in the Sino-Vietnamese border region [4]. Previous studies mainly focused on taxonomic revisions, such as the confirmation of the taxonomic status of *Lysionotus pterocaulis* (C. Y. Wu ex W. T. Wang) H. W. Li [11]. In recent years, transcriptome-based biogeographic analyses have revealed that *Lysionotus* originated from the karst regions spanning northern Vietnam, southeastern Yunnan, southwestern Guangxi, and southern Guizhou. The divergence of most species was primarily driven by Miocene climate fluctuations, leading to eastward and westward range expansions, which formed the current continuous distribution pattern from northern India through southern China to southern Japan [12]. However, studies on plastid genome structural variations adaptability remain unreported.

*Anna* Pellegr. [13] includes four published species [14,15]. These subshrubs are predominantly distributed in limestone hills along the Sino-Vietnamese border, extending to Guangxi, Yunnan, Guizhou, Sichuan (China) and northern Vietnam. These species typically inhabit shaded dense forests on mid-to-high altitude slopes and limestone habitats and are distinguished by their large bracts and zygomorphic flowers. The taxonomic status of *Anna* has long been controversial: traditional morphology classified it under Trib. Didymocarpeae [16,17], but recent molecular phylogenetic studies have supported monophyletic *Lysionotus* and *Anna* as sister groups [12], both under Trib. Trichosporeae. Thus, an exhaustive systematic genomic investigation of all species within genus *Anna* is urgently required to elucidate their evolutionary and phylogenetic relationships.

*Lysionotus* and *Anna*, as closely related genera of Gesneriaceae [12], are typical endemic plants in the karst habitats along the Sino-Vietnamese border [4]. The phylogenetic relationship between *Anna* and *Lysionotus* has been gradually clarified by classification and molecular evidence across different periods: A 1995 classification placed the two genera in different tribes within the subfamily Cyrtandroideae, with *Anna* assigned to Trib. Didymocarpeae and *Lysionotus* to trib. Trichosporeae [18]. Weber grouped both genera into the “Advanced Asiatic and Malesian genera” of Didymocarpoid Gesneriaceae in 2004 [19]. In 2011, molecular phylogenetic studies based on ITS and *Trnl-F* by Moller confirmed a close relationship between the two genera, with both forming well-supported monophyletic clades [20]. They share a synapomorphy of seeds with appendages at both ends, hence were classified in trib. Trichosporeae, with the key morphological distinction being stamen number (four in *Anna* vs. two in *Lysionotus*) [20,21]. This conclusion is consistent with the framework of *Anna* and *Lysionotus* being sister groups based on transcriptome data, further validating their close phylogenetic affinity [12].

The systematic framework of Gesneriaceae has undergone in-depth revision and improvement in recent years, underpinned by combined evidence from molecular systematics and morphology, with the delimitation criteria and evolutionary affinities of core clades further clarified [22,23]. In tribe Trichosporeae, a comprehensive analysis of nine DNA fragments and morphology revealed the polyphyly of traditional genera [24]. Key revision progress has been made in *Didymocarpus*, *Allocheilos* and the subtribe Leptoboeinae [25,26,27]. There are no revision records for *Lysionotus* and *Anna*, and they maintain stable systematic positions under Trichosporeae.

As a maternally inherited molecular marker, the chloroplast genome is characterized by structural stability (approximately 120–160 kb) and a moderate evolutionary rate [28,29,30], which has been widely used in plant phylogenetic reconstruction [31,32], estimation of species divergence times [33,34] and identification of adaptively evolving genes [35,36]. In particular, the variation patterns of its coding regions (e.g., *rbcL*, *matK*) and non-coding regions (e.g., *trnH-psbA*) can effectively reveal phylogenetic relationships among closely related genera and adaptive selection [37,38,39]. However, comparative studies on the chloroplast genomes of endemic *Lysionotus* and *Anna* plants along the Sino-Vietnamese border have not been conducted, which limits our understanding of the evolutionary history and ecological adaptation mechanisms of these two genera in this region.

The objectives of this research were threefold: (1) to compare the structural characteristics of the chloroplast genomes between the two genera, including gene order, repeat sequence distribution and variations in inverted repeat (IR) region boundaries; (2) to reconstruct the phylogenetic tree based on whole chloroplast genome data and coding sequences (CDs), thereby clarifying the phylogenetic relationships between the two genera; (3) to elucidate the adaptive evolutionary patterns of these species in the unique karst habitats of the Sino-Vietnamese border region. The results of this study will provide molecular insights into the evolution of different sympatrically distributed species and offer a genomic basis for the conservation of endemic plant resources.

## 2. Materials and Methods

### 2.1. Plant Materials and DNA Extraction

A total of 10 new plastomes were obtained for this study: four represented all *Anna* species, and the remaining 6 were *Lysionotus* species distributed in the border area between China and Vietnam (Table 1). To improve the reliability of phylogenetic inference, the datasets comprise three components: (1) published chloroplast genomes of trib. Trichosporeae and trib. Didymocarpeae retrieved and filtered from the public National Center for Biotechnology Information (NCBI) database, with one representative chloroplast genome selected per genus to ensure taxonomic representativeness; (2) 24 chloroplast genomes of other *Lysionotus* species also downloaded from public databases; (3) ten newly obtained complete chloroplast genomes generated in this study. *Petrocodon jingxiensis*, *Primulina linearifolia*, *Didymocarpus yuenlingensis*, *Hemiboea pterocaulis*, *Oreocharis burtii*, *Henckelia pumila* and *Boeica multinervia* were selected as outgroups. Thus, a total of 46 complete chloroplast genomes were employed for phylogenetic reconstruction (detailed information regarding the public data is provided in Table S1).

Species identification was based on the latest research literature on *Lysionotus* and *Anna* and detailed morphological descriptions of two genera in flora of China, combined with our careful observations on important identification characteristics (including plant habit, leaf shape, floral organs and fruits) to ensure species accuracy. Fresh leaf tissue was immediately preserved in silica gel and the total DNA was extracted with the NuClean Plant Genomic DNA Kit from CWBIO. After extraction, the quality of the DNA was evaluated by 1.0% (*w*/*v*) agarose gel electrophoresis stained with SYBR Green I and a Nanodrop2000 (Thermo Fisher Scientific, Waltham, MA, USA) to assess the concentration and purity. Then, the DNA library was constructed at 250 to 300 bp, with DNA fragments converted into Illumina high-throughput sequencing using the Illumina Nova6000 Platform (Illumina, San Diego, CA, USA), producing a total of 6 Gb of raw data for each sample.

### 2.2. Chloroplast Genome Sequencing, Assembly and Annotation

We constructed a DNA library with 300 bp inserted fragments following the Ilumina NovaSeq protocol for each sample. The raw sequencing image data were converted into sequence data saved in the FASTQ format. The high-throughput sequencing was per- formed on the Illumina Nova6000 Platform (Illumina, San Diego, CA, USA) and produced about 6 Gb of raw data for each sample. The clean reads were assembled using GetOrganelle v1.7.5 [40] with default parameters. The assembled genomes were checked and visualized in Bandage v0.7.1 [41]. Finally, the plastomes were annotated in Geseq [42] and manually checked with *Loxostigma kurzii* (NC_082151.1) as reference. More than one closely related species was selected as an annotation reference, whereas others either contained abnormal introns or lacked CDs that could be aligned in NCBI. Therefore, NC_082151.1, with relatively accurate and complete annotation, was chosen as the reference genome for correction. The physical maps were drawn using OGDRAW v1.3.1 [43]. The genome length and GC content were calculated using SeqKit [44], and the numbers of CDs, transfer RNAs (tRNAs) and ribosomal RNAs (rRNAs) in the annotated chloroplast genome were statistically analyzed with Geneious Prime 2025 [45].

### 2.3. Comparative Genomic Analysis and Nucleotide Diversity

To elucidate the divergence within the chloroplast genomes, we used the Perl script (available at https://github.com/quxiaojian/Bioinformatic_Scripts, accessed on 11 December 2022) to transform the GenBank files into mVISTA-compatible formats. We compared the complete chloroplast genome sequences of the 10 new chloroplast genomes using mVISTA (https://genome.lbl.gov/vista/mvista/submit.shtml; accessed on 3 April 2023) in Shuffle-LAGAN mode [46]. To analyze the expansion and contraction of the IR regions, the CPJSdraw v1.0.0 [47] software was employed to delineate the boundaries between the single-copy (SC) and IR regions.

To identify hypervariable regions in the chloroplast genome for future deeply genetic population and species identification studies, sliding window analysis was conducted in DnaSP v.6.12.03 [48] and nucleotide diversity (Pi) was calculated across whole chloroplast genome regions. The analysis was conducted on an alignment of 10 chloroplast genomes which were aligned in MAFFT v.7 [49]. The sliding window width was set to 500 bp and the step size was also set to 500 bp.

Repetitive sequences represent prominent genomic features that shape genome evolution, inheritance, and genetic variation. SSRs were detected using MISA (https://webblast.ipk-gatersleben.de/misa/; accessed on 21 March 2023) [50]. SSRs were defined as motifs with unit sizes ranging from mononucleotides to hexanucleotides, with the minimum number of repeats required for identification set to 10, 5, 4, 3, 3 and 3, respectively. This parameterization was based on the inherent characteristics of SSR motifs; short motifs (e.g., mononucleotides) exhibit higher inherent randomness, thus necessitating more repeats to form stable SSR structures. In contrast, long motifs (e.g., tetranucleotides to hexanucleotides) possess greater specificity and are more prone to forming functional SSRs even with fewer repeats, so the minimum repeat threshold was appropriately reduced for these motifs. The resulting SSR datasets were processed and visualized using Microsoft Excel.

REPuter (https://bibiserv.cebitec.uni-bielefeld.de/reputer/; accessed on 30 March 2023) [51] was employed to identify large repeat sequences, including the size and genomic location of forward, reverse, complement and palindromic repeats. The Hamming distance was set to 3, as this parameter not only captures genuine repetitions that may harbor natural variations but also mitigates false positives, thus balancing the sensitivity and specificity of the analysis. Additionally, the analysis was constrained to a maximum of 5000 repeat sequence sets, as this constraint ensures computational efficiency while precluding the omission of authentic long repeats naturally present in chloroplast genomes. To exclude sequences shorter than 30 bp, the minimum repeat size was set to 30. This threshold was selected because large repeat sequences (≥30 bp, such as dispersed long repeats) were key elements modulating the structural stability and evolutionary recombination of genomes.

### 2.4. Adaptive Evolution Analysis

Adaptive evolution analysis, encompassing codon usage and selection pressure analyses, was conducted based on protein-coding genes (PCGs) to characterize their adaptive evolution from both codon bias and selection pressure aspects. First, the common single-copy coding sequences were extracted by PhyloSuite v1.2.2 [52], with sequences of less than 300 bp in length and those initiating with non-ATG start codons; sequences with abnormal stop codons and those containing internal stop codons within the CDs were excluded, with the remaining CDs used for analysis. For the analysis of codon usage, Perl scripts were used to quantitatively calculate codon-related indices for the filtered sequences. The specific indices included: the total number of codons, base composition-related indices (GC content at the first position of codons (GC1), GC content at the second position (GC2), GC content at the third position (GC3), and average GC content of the entire sequence (GC_all_average)), codon preference evaluation indices (effective number of codon (ENC); relative synonymous codon usage (RSCU)), and GC content at the third position of synonymous codons (GC3s). Based on the obtained data, statistical analysis and visualization were performed with the aid of R (v 4.3.2) [53] and related packages to achieve intuitiveness of the results. In addition, stacked plots were constructed using the ggplot2 package [54] to analyze the distribution differences of codon indices among different functional gene subsets, assisting in locating preference characteristics.

To detect the selective pressures acting on protein-coding genes, the branch-site model in the CODEML program of PAML v 4.10.7 [55] was employed to estimate the nonsynonymous (dN) and synonymous (dS) substitution rates, as well as their ratio (w = dN/dS). First, single-copy CD sequences were extracted using Phylosuite v1.2.2 software, then aligned by projecting onto corresponding amino acid alignments generated using MAFFT v.7. Subsequently, maximum likelihood phylogenetic trees were constructed based on the concatenated CDs using IQ-TREE v1.2.2 [56]. As ω > 1 indicates positive selection, ω = 1 denotes neutral evolution and ω < 1 suggests purifying selection [57,58,59]. Log-likelihood values were calculated under an alternative branch-site model (Model = 2; NSsites = 2; Fix = 0), allowing ω to vary among codons along target branches, and a null model (Model = 2; NSsites = 2; Fix = 1; Fixω = 1). Likelihood ratio tests (LRTs) were performed by comparing these models, with statistical significance assessed via a one-degree-of-freedom right-tailed chi-square test [60]. Genes with adjusted *p*-values < 0.05 and identified positively selected sites were designated as positively selected genes [61]. Furthermore, the Bayesian Empirical Bayes (BEB) approach was employed, which estimated the posterior probabilities of each codon belonging to distinct selective pressure regimes [62]. Codon sites with high posterior probabilities (typically ≥0.95 as a widely accepted threshold in selection pressure analyses) were designated as positively selected sites (PSSs) [62,63].

### 2.5. Phylogenetic Analysis

We obtained 10 complete chloroplast genomes, with single-copy common CDs shared across species also extracted to determine the phylogenetic position of *Lysionotus* and *Anna* species. Phylogenetic trees were constructed using the maximum likelihood (ML), Bayesian inference (BI), and neighbor-joining (NJ) methods based on two datasets (whole chloroplast genome datasets and chloroplast CDs datasets). Multiple sequence alignment was performed using MAFFT v.7 with the auto option. The optimal nucleotide substitution model was selected using jModelTest v2.1.10 [64], and the results indicated the GTR+I model was the best-fit model for both maximum likelihood (ML) and Bayesian inference (BI) methods for CD datasets and the GTR+F+I+G4 model was the best-fit model for the two methods for complete chloroplast genome datasets, which were then automatically optimized. To evaluate the reliability of each branch in the tree topology, 5000 replicates of the ultrafast bootstrap (UFBoot) test were performed to correct for biases in IQ-TREE v1.2.2.

MrBayes v 3.2.7 [65] was employed with the following settings: Nst = 6; the prior for substitution rate ratios was Dirichlet (1.00, 1.00, 1.00, 1.00, 1.00, 1.00); rates = Invgamma with Ngammacat = 4; the prior for Alpha was an exponential distribution (1.00); the prior for Pinvar was a uniform distribution over the interval (0.00, 1.00); and Statefreqpr = Fixed (Empirical). The MCMC algorithm utilized multiple movement strategies to improve sampling efficiency. The total number of generations was set to 2,000,000 to ensure the chains reached a stable state sufficiently. Four parallel chains (3 heated chains and 1 cold chain) were run independently twice simultaneously to avoid local optima, with the heating temperature set to the default value of 0.2. Sampling and result printing were conducted every 1000 generations, and a checkpoint was set every 5000 generations. A relative burn-in period of 25.0% was adopted, discarding the first 500,000 generations of samples. Convergence was assessed via multi-dimensional indicators, with the Average Standard Deviation of Split Frequencies (ASDSF) as the core metric. The variation trend of log-likelihood values was also considered, and auxiliary validation was performed via Tracer v1.7.1 [66] to ensure evaluation rigor. The Effective Sample Size (ESS) of all key parameters exceeded 200. After meeting the convergence criteria, phylogenetic tree construction and parameter estimation were completed based on the qualified samples.

A neighbor-joining (NJ) phylogenetic tree was constructed using MEGA v.11 [67] and the parameters were set; a 5000-replicate Bootstrap analysis was conducted to assess node reliability, and the test type was set to Bootstrap consensus tree to enhance the robustness of the results. The Kimura 2-parameter (K2P) model was prioritized as the substitution model to account for the differences between base transitions and transversions. All resulting phylogenetic trees were visualized using the Interactive Tree of Life (iTOL) v6 software [68]. The visualization process included integrating bootstrap support values and posterior probabilities. Tree layout adjustments (e.g., branch length scaling, clade coloring, tip label formatting) were performed in iTOL to enhance readability and clarity of evolutionary relationships.

## 3. Results

### 3.1. Comparison of Organelle Genome Characteristics

**Figure 1:**
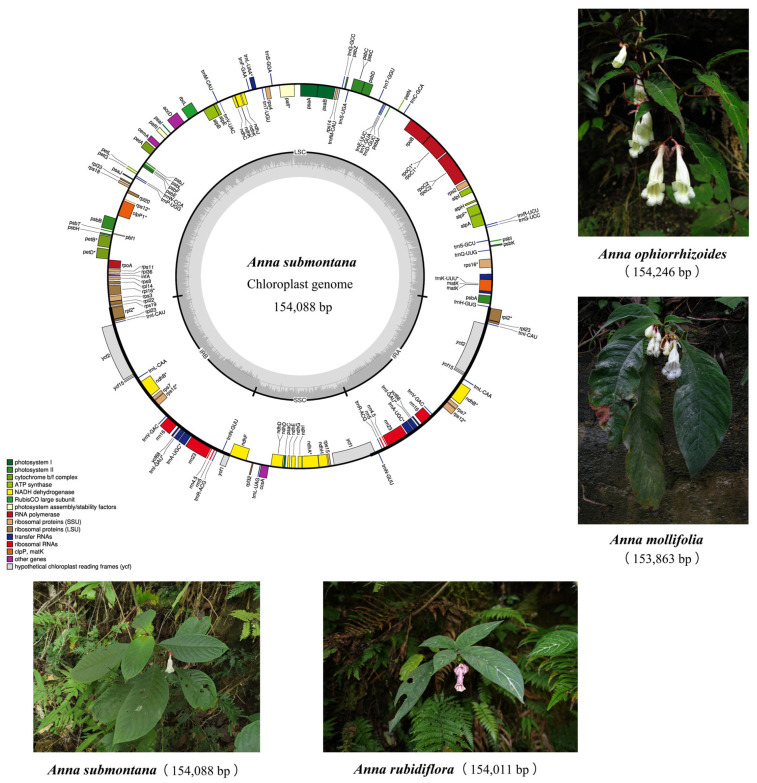
Chloroplast genome map of Anna species generated in this study. The genes inside the outer circle are transcribed clockwise while the outside genes are transcribed anti-clockwise. Genes are color-coded according to their functional groups. The darker gray columns in the inner circle denote the GC content across the genome and the lighter gray columns, accordingly, correspond to the AT content; * indicates that the gene contains an intron. Morphological characteristics of four species generated in this study are also illustrated.

Ten chloroplast genomes, representing different species from two closely related genera and distributed in the Sino-Vietnamese border region, were compared in this study. The chloroplast genomes of four Anna species exhibited highly conserved characteristics. The total chloroplast genomes ranged from 154,011 to 154,246 bp and the overall GC content was highly stable, varying narrowly from 37.44% to 37.49%. In terms of genomic structure, both length and GC content of each region showed variations: (1) The length of large single-copy (LSC) ranged from 84,959 to 85,104 bp and GC content varied from 35.37% to 35.42%; (2) the small single-copy (SSC) length spanned 18,283–18,360 bp and GC content ranged from 31.04% to 31.19%; (3) the inverted repeat (IR) region had the smallest length variation (25,359–25,396 bp) and the GC content was the most stable (43.20–43.23%). The complete chloroplast genome map of Anna species is presented in Figure 1.

For the six *Lysionotus* chloroplast genomes, lengths ranged from 154,184 to 154,278 bp and the overall GC content varied slightly, ranging from 37.48% to 37.58%. LSC lengths spanned from 85,047 to 85,144 bp and GC content ranged from 35.42% to 35.54%. In terms of SSC, lengths varied from 18,199 to 18,250 bp and GC content ranged from 31.18% to 31.38%. For IR regions, lengths ranged narrowly from 25,444 to 25,467 bp and GC content was relatively conserved, varying from 43.17% to 43.21%. Notably, *L. chingii* consistently showed the lowest GC content across all four genomic regions (complete plastome, LSC, SSC and IR), which was distinct from other *Lysionotus* species. This observation suggested that *L. chingii* has a stronger preference for AT-rich sequences compared to its congeneric counterparts. Collectively, the length variation in these 10 chloroplast genomes was primarily driven by differences in LSC. Additionally, no large-fragment deletions were detected in the IR regions of all 10 chloroplast genomes tested, and the two IR regions within each genome showed identical lengths, confirming the extremely high structural conservatism of the IR region in Anna and *Lysionotus* chloroplast genomes.

**Figure 2:**
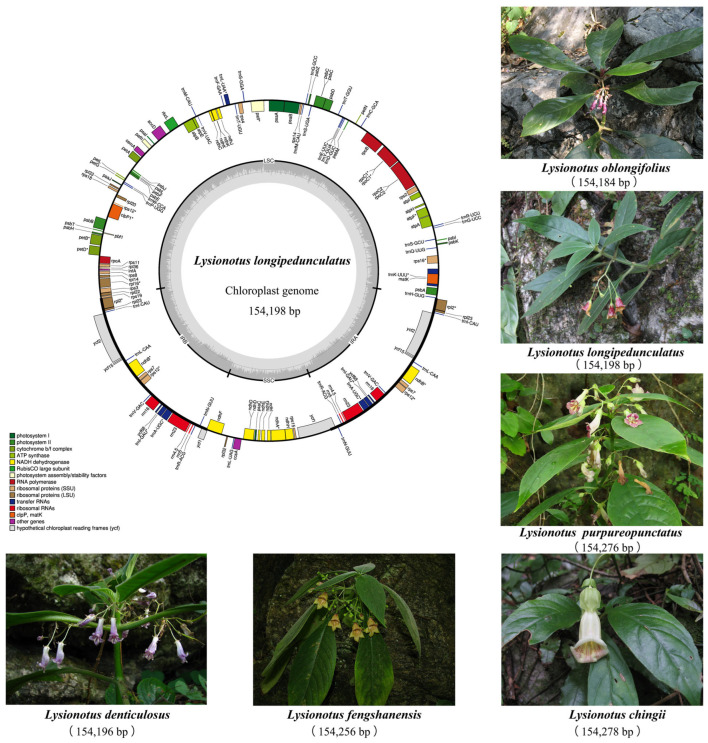
Chloroplast genome map of *Lysionotus* species generated in this study. The genes inside the outer circle are transcribed clockwise while the outside genes are transcribed anti-clockwise. Genes are color-coded according to their functional groups. The darker gray columns in the inner circle denote the GC content across the genome and the lighter gray columns, accordingly, correspond to the AT content; * indicates that the gene contains an intron. Morphological characteristics of six species generated in this study are also illustrated.

The statistics of genes from 10 chloroplast genomes revealed high conservatism of ribosomal RNA (rRNA) genes, and all individuals consistently harbored eight rRNA genes. However, differentiation was observed in the number of CDs and transfer RNA (tRNA) genes between the two genera. The variation number in CDs ranged from 85 to 88, and the tRNA gene number ranged from 36 to 38 in Anna. All six *Lysionotus* species stably contained 87 CDs without intraspecific variation. Although variation in the tRNA gene number existed, its amplitude was lower than that in Anna. Only *L. denticulosus* harbored 36 tRNA genes, while the other five species all contained 35 tRNA genes. The characteristics of the 10 chloroplast genomes are shown in Table 2. The complete chloroplast genome map of *Lysionotus* species is presented in Figure 2.

### 3.2. IR Contraction and Expansion Analysis

**Figure 3:**
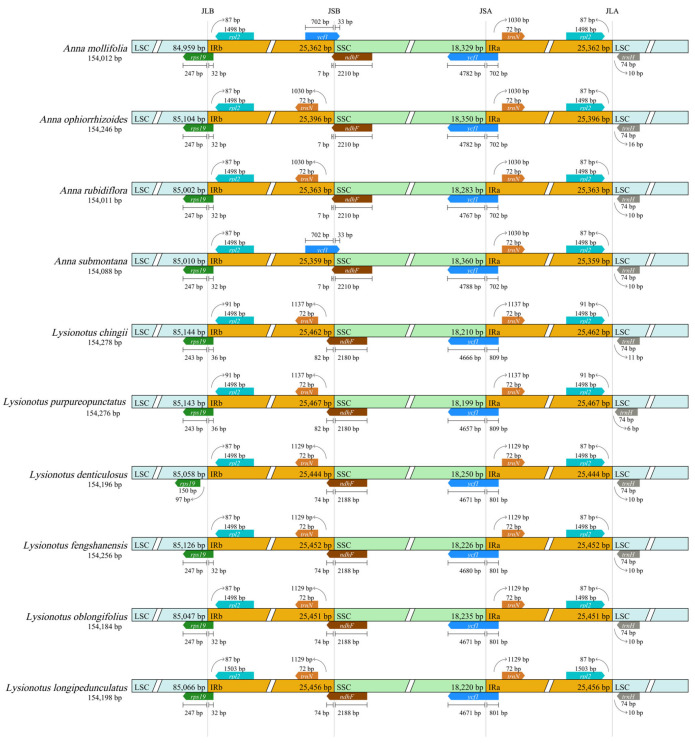
Visualization results of boundary analysis among 10 chloroplast genomes (based on CPJSdraw). Blue, green, and orange represent LSC, SSC, IRa/IRb, respectively; arrows indicate the transcription direction of genes adjacent to the boundaries, with gene names labeled.

All samples maintained the typical quadripartite structure (LSC-IRb-SSC-IRa) and the arrangement of core boundary genes exhibited conservation (Figure 3). Two IRs in the same chloroplast genome were identical in length and no significant expansion or contraction was observed in the two genera. JLB, JSB, JSA and JLA correspond to the boundaries between LSC-IRb, IRb-SSC, SSC-IRa and IRa-LSC, respectively. Genes adjacent to these boundaries included *rps19*, *rpl2*, *ycf1*, *ndhF*, *trnN* and *trnH.* The *Rps19* gene spanned the boundaries of LSC and IRb in nine samples, with the exception of *L. denticulatus*, which did not cross this boundary. *Rpl2*, as another boundary-associated gene near JLB, was located entirely within IRb in all samples. Within *Anna*, the length of *rpl2* was consistently 1498 bp and 87 bp away from JLB. Among *Lysionotus* species, *L. longipedunculatus* had the longest rpl2 length (1503 bp), which was also 87 bp away from JLB. *L. denticulatus*, *L. oblongifolius* and *L. fengshanensis* all had an *rpl2* length of 1498 bp, with an 87 bp distance from JLB. Both *L. chingii* and *L. purpureopunctatus* possessed 1498 bp *rpl2*, which was 91 bp away from JLB.

In *A. mollifolia* and *A. submontana*, *ycf1*, as a boundary-associated gene near both JSB and JSA, spanned JSB and resulted in 702 bp fragments in IRb and 33 bp fragments in SSC. For the other eight samples, no *ycf1* was detected; rather, 72-bp *trnN* was identified near JSB and did not cross. The *ndhF* spanned JSB in all samples and consisted of 2217 bp in *Anna* species, with 76 bp in IRb and 2210 bp in SSC. In *Lysionotus* species, the *ndhF* length was uniformly 2262 bp, *L. denticulatus*, *L. longipedunculatus*, *L. oblongifolius* and *L. fengshanensis* had 74 bp in IRb and 2188 bp in SSC, while *L. chingii* and *L. purpureopunctatus* contained 82 bp in IRb and 2180 bp in SSC.

*Ycf1* spanned JSA in all 10 samples and the segment in IRa was relatively conserved, with differences in total length primarily attributed to variations in SSC. Within *Anna*, the *ycf1* segment in IRa was consistently 702 bp, with *A. mollifolia* and *A. ophiorrhizoides* having 4782 bp in SSC, *A. rubriflora* containing 4767 bp in SSC, and *A. submontana* having 4788 bp in SSC. Within *Lysionotus*, *L. denticulatus*, *L. longipedunculatus*, *L. oblongifolius* and *L. fengshanensis* shared 801 bp in IRa, with 4671 bp, 4671 bp, 4671 bp, and 4680 bp in SSC, respectively. *L. chingii* and *L. purpureopunctatus* had 809-bp in IRa, and their segments in SSC contained 4666 bp and 4657 bp.

The *rpl2* gene was exclusively localized in IRa across all samples and did not span JLA, with a distance of 87–91 bp from the boundary. Within *Anna*, *rpl2* exhibited a consistent 1498 bp and kept 87 bp to JLA. Within *Lysionotus*, variations were observed in *rpl2* length and in its distance to JLA. The *trnH* maintained a consistent 74 bp across all 10 samples, with a distance of 6–16 bp from JLA. Within *Anna*, only *A. ophiorrhizoides* had 16 bp away from JLA, with the remaining *Anna* species showing 10 bp distance from JLA. Within *Lysionotus*, *L. denticulatus*, *L. longipedunculatus*, *L. oblongifolius* and *L. fengshanensis* kept 10 bp away from JLA. *L. chingii* was 11 bp away from the boundary, and *L. purpureopunctatus* had the shortest distance of 6 bp.

### 3.3. Highly Variable Regions and Repetitive Element Analysis

**Figure 4:**
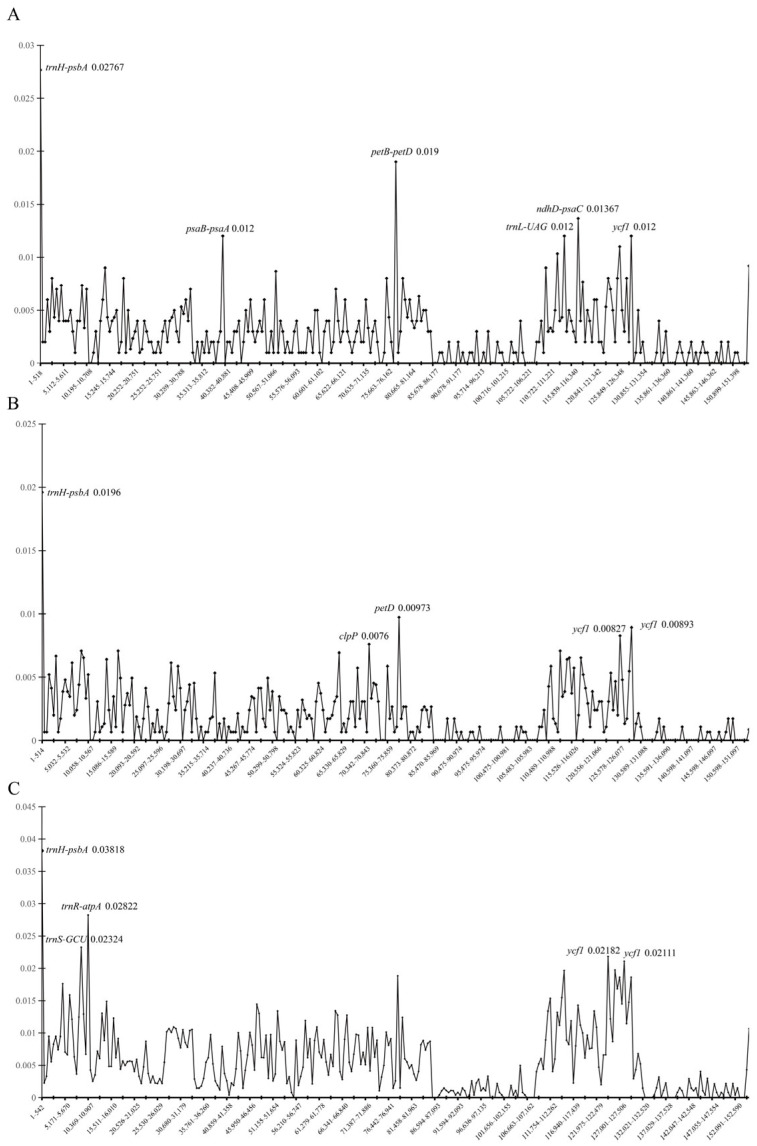
Nucleotide diversity based on the entire chloroplast genomes of *Anna* and *Lysionotus* species (window length: 500 bp; step size: 500 bp). The X-axis shows the position of each window; the Y-axis shows the nucleotide diversity (pi) of each window. (**A**) The chloroplast genome Pi of four *Anna* species; (**B**) the chloroplast genome Pi of six *Lysionotus* species; (**C**) the Pi of 10 chloroplast genomes.

Based on the standard thresholds of the genetic diversity index (Pi) (low genetic diversity ≤ 0.005, moderate genetic diversity 0.005~0.02, high genetic diversity > 0.02), the variation characteristics of chloroplast fragments in the studied species are as follows (Figure 4):

In *Anna*, *trnH-psbA* (Pi = 0.02767) was a high genetic diversity fragment and the most significantly variable fragment in this genus; *petB-petD* (Pi = 0.019), *ndhD-psaC* (Pi = 0.01367), *trnL*-UAG (Pi = 0.012), *psaB-psaA* (Pi = 0.012), and *ycf1* (Pi = 0.012) all showed moderate genetic diversity. The overall genetic diversity of the chloroplast genome in this genus was moderately high. Among the six species of *Lysionotus*, *trnH-psbA* (Pi = 0.0196) had moderate genetic diversity and was the most variable fragment in this group; *petD* (Pi = 0.00973), *clpP* (Pi = 0.0076), and *ycf1* (Pi = 0.00827, 0.00893) all exhibited low to moderate genetic diversity (close to the 0.005 threshold). The overall variation level of the chloroplast genomes of these six species was relatively low, with only a few fragments reaching moderate diversity. Among the 10 chloroplast genomes, multiple fragments were high genetic diversity hotspots: *trnH-psbA* (Pi = 0.03818) had the most significant variation, while *trnR-atpA* (Pi = 0.02822), *trnS*-GCC (Pi = 0.02324), and *ycf1* (Pi = 0.02182, 0.02111) all showed high genetic diversity.

Consistent with Pi results, mVISTA alignments (using *L. longipedunculatus* as the reference) validated genome-wide genetic diversity (Figure S1). Alignments of 10 chloroplast genomes showed sequence similarity ranging from 50% to 100%, core functional genes were 100% conserved, whereas most genes and IGSs only reached 50% similarity, corresponding directly to the high Pi values in these regions. Additionally, the number of aligned regions varied interspecifically (e.g., 257 in *A. rubidiflora* vs. 268 in *A. ophiorrhizoides*), further reflecting interspecific differentiation.

### 3.4. Quantification of Simple and Large Repeat Sequences

**Figure 5:**
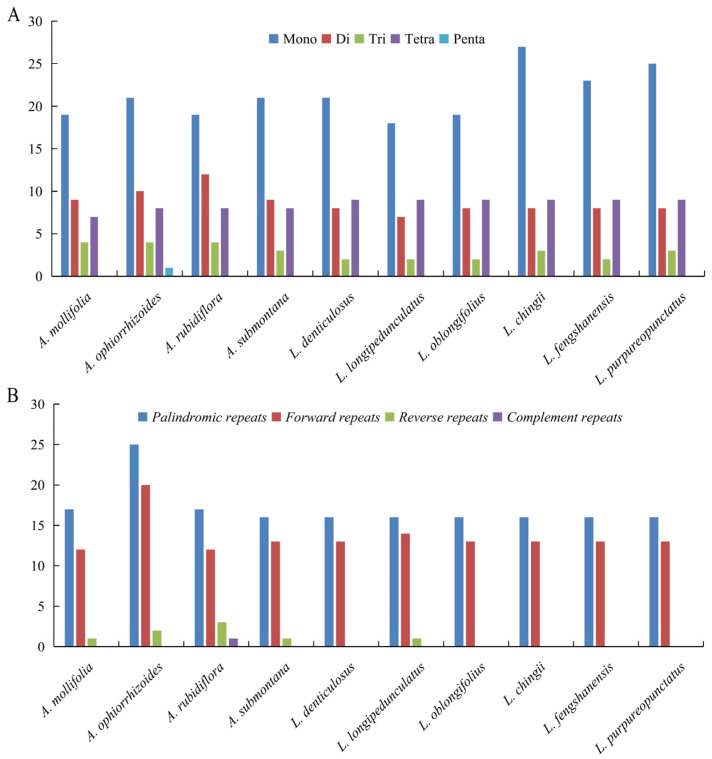
Statistics of sequence repeats in 10 chloroplast genomes. (**A**) Types and numbers of SSRs in 10 chloroplast genomes. (**B**) Comparison of large repeat sequences in 10 chloroplast genomes.

Five types of SSRs were identified across the 10 chloroplast genomes investigated in this study. Among *Anna*, the total number of SSRs ranged from 39 to 44, with *Lysionotus* species exhibiting a total SSR count spanning from 36 to 45. *Anna* species exhibited greater diversity in SSR types, encompassing five repeat categories: mononucleotide, dinucleotide, trinucleotide, tetranucleotide and pentanucleotide repeats. It should be emphasized that pentanucleotide repeats were detected exclusively in *A. ophiorrhizoides* from examined *Anna*. In contrast, all six *Lysionotus* species contained four SSR repeat types (mononucleotide, dinucleotide, trinucleotide, and tetranucleotide), though SSR counts differed across species. Across both genera, mononucleotide repeats exhibited the most pronounced interspecific variation (Figure 5A). Within *Anna*, mononucleotide repeat counts ranged from 19 to 21; *A. mollifolia* and *A. rubriflora* had the minimum (19), whereas *A. ophiorrhizoides* and *A. submontana* had the maximum (21). For *Lysionotus*, mononucleotide repeat counts spanned a wider range (18–27), with *L. longipedunculatus* exhibiting the minimum (18) and *L. chingii* the maximum (27).

Large repeat sequences (LRSs) in *Anna* consisted of palindromic repeats (P), forward repeats (F), reverse repeats (R) and complementary repeats (C). Palindromic repeats ranged from 16 to 25 (Figure 5B), with *A. submontana* showing the lowest threshold and *A. ophiorrhizoides* the highest. Forward repeats ranged from 12 to 20, with *A. rubriflora* having the lowest threshold and *A. ophiorrhizoides* the highest. Reverse repeats ranged from 1 to 3, with both *A. mollifolia* and *A. submontana* possessing one reverse repeat, while *A. rubriflora* exhibited the highest threshold. Only one complementary repeat was detected, exclusively in *A. rubriflora*. Six *Lysionotus* species contained only three types of LRSs, with no complementary repeats detected. There were consistently 16 palindromic repeats across all species. There were 14 forward repeats were in *L. longipedunculatus*, while the remaining *Lysionotus* species all possessed 13. One reverse repeat appeared exclusively in *L. longipedunculatus*. Overall, conservatism in types and quantities of LRSs was observed among the Sino-Vietnamese border *Lysionotus* species.

### 3.5. Codon Usage Analysis

**Figure 6:**
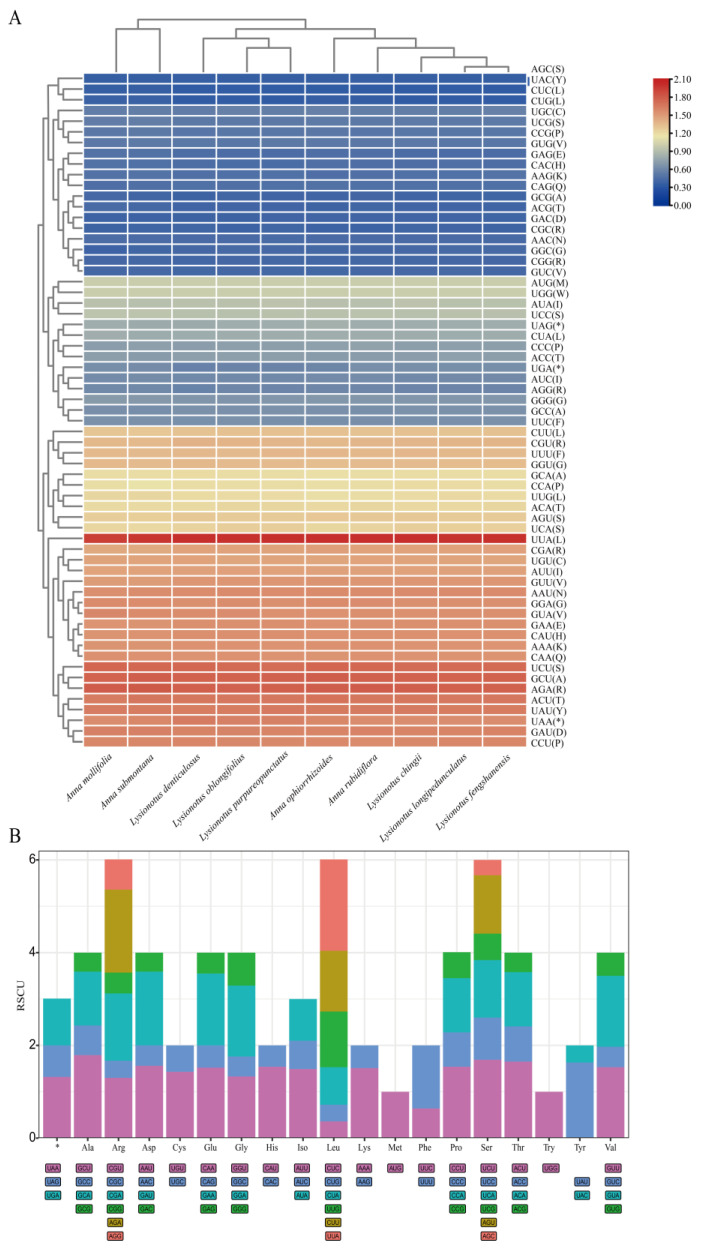
The RSCU of 79 coding sequences in the 10 samples represented. (**A**) The species–codon heatmap of the RSCU of 64 codons across 10 chloroplast genomes. Red boxes imply highly preferred synonymous codons (RSCU > 1); blue boxes indicate less preferred synonymous codons (RSCU < 1). (**B**) The average RSCU of 10 chloroplast genomes. Each category on the x-axis corresponds to a single amino acid, and differently colored bars within the same amino acid category represent distinct synonymous codons, with column height corresponding to the RSCU of the respective codon.

To assess the potential impact of codon usage on gene expression and evolutionary adaptation, we analyzed the codon usage frequency and relative synonymous codon usage (RSCU) for protein-coding genes in *Anna* and *Lysionotus* species chloroplast genomes (Figure 6). Figure 6A is a species–codon heatmap constructed using RSCU, which captures interspecific differences and preferences in codon usage patterns. Clustering analysis of the heatmap revealed convergent codon usage preferences among *Anna* and *Lysionotus* species distributed along the China–Vietnam border, with these lineages showing predominant bias toward the codons UUA, UCU, GCU, and AGA.

Figure 6B illustrates amino acid–synonymous codon usage preferences, which visualizes the relative usage frequencies of synonymous codons for each amino acid (including stop codons marked as *). The three stop codons (UAA, UAG, UGA) exhibited low comparable RSCU, indicating relatively balanced usage with no significant preference for any specific stop codon. For amino acids encoded by a single codon (i.e., AUG for methionine and UGG for tryptophan), their RSCU values directly reflect the actual usage frequencies of these unique codons. Leucine (Leu), arginine (Arg) and serine (Ser) displayed the highest total RSCU values (~6), signifying frequent usage of their synonymous codon. Among the synonymous codons for Leu (e.g., CUC, CUG, CUA), UUA had markedly higher RSCU value than the others (orange in Figure 6B), confirming it as a highly preferred codon (consistent with the heatmap); CUU was also identified as a preferentially used codon for Leu. For Arg, three synonymous codons (CGU, CGA, AGA) had RSCU values > 1, indicating their preferential selection. Ser amino acids exhibited preferential usage of UCU, UCA and AGU, all with RSCU values > 1. In addition, aspartic acid (Asp), glutamic acid (Glu), and glycine (Gly) each had multiple codons with RSCU values > 1, further demonstrating biased codon usage for these amino acids. Collectively, the stacked plot confirmed *Anna* and *Lysionotus* species along the China–Vietnam border region exhibit significant codon usage bias, with most amino acids preferentially encoded by specific synonymous codons (corresponding to bars with high RSCU values), a pattern that reflects the non-random selection of codons during the process of gene expression in these lineages.

Furthermore, the GC contents at different codon positions within the coding sequences (CDs) of the organellar genomes were calculated (Table 3). The empirical values of effective codon number (ENC) corresponding to GC content at the third synonymous codon (GC3) exhibited a consistent species-wide deviation pattern upon fitting to the standard curve, which implied that synonymous codon usage bias in these species was constrained and shaped by natural selection pressure (Figure S2). PR2-plot analysis demonstrated that the base preferences of codons were not clustered around the PR2 equilibrium point (0.5, 0.5) and were instead distributed across four quadrants (Figure S3). The base composition deviated from PR2 parity, suggesting codon usage bias among individual genes was predominantly shaped by natural selection. Our results also revealed divergence in codon usage profiles across genes of distinct functional categories. Neutral analysis revealed the scatter points exhibited no linear clustering (Figure S4). Regression modeling yielded slope values ranging from 0.16 to 0.224 (~0), demonstrating that the GC content at the third codon position (GC3) had extremely limited explanatory power for variations in the GC content at the first and second codon positions (GC12). Additionally, the adjusted coefficient of determination (R2adj) was nearly zero, indicating an extremely poor model fit and confirming the absence of a meaningful linear correlation between GC3 and GC12. Collectively, ENC-plot, PR2-plot and neutral analysis consistently supported the dominance of natural selection in shaping the codon usage patterns of these species.

### 3.6. Selection Pressure Analysis

A total of 79 shared single-copy genes were extracted for selection pressure analysis based on 10 chloroplast genomes. According to ω value (nonsynonymous substitution rate/synonymous substitution rate) and statistical significance, these genes were categorized into three groups (Table S2): genes with significant signals consistent with positive selection (2), genes with potential signals consistent with positive selection (44), and neutral/negatively selected genes (33). These species distributed along the Sino-Vietnamese border generally exhibited a conservative evolutionary trend, with most genes showing no signals consistent with positive selection (ω < 1) and being significantly subjected to purifying selection. This indicated that the nucleotide sequences and the biological functions of their encoded proteins were highly conserved, while statistical results demonstrated that only *ycf1* and *atpH* showed significant signals consistent with positive selection (ω > 1 and *p* < 0.05), providing key clues for deciphering the species’ habitat adaptation. Specifically, the *ycf1* gene is primarily involved in stress response and transmembrane substance transport. Its relatively high ω value may be closely linked to the functional differentiation of species adapting to the special stressful habitats (e.g., extreme climates, soil stress) in the Sino-Vietnamese border region. Signals consistent with positive selection with respect to *atpH* may reflect the capacity for energy utilization in specific habitats, thereby enhancing survival competitiveness, as this gene participates in the ATP synthesis-related energy metabolism pathway.

Notably, besides aforementioned significantly positively selected genes, some genes exhibited ω > 1 but *p* ≥ 0.05, failing to reach statistical significance and showing potential positive selection tendency, thus defined as potentially positively selected genes. Residing in the unique geographical and climatic conditions of the Sino-Vietnamese border region, these positive selection signals provide a critical genetic variation basis for the species to adapt to this distinctive habitat. The accumulation of nonsynonymous substitutions can promote gene functional differentiation and species adaptive evolution. In addition, these positively selected genes may be involved in regulating the formation of key traits in terms of adapting to specific habitats.

### 3.7. Molecular Phylogenies Analysis

**Figure 7:**
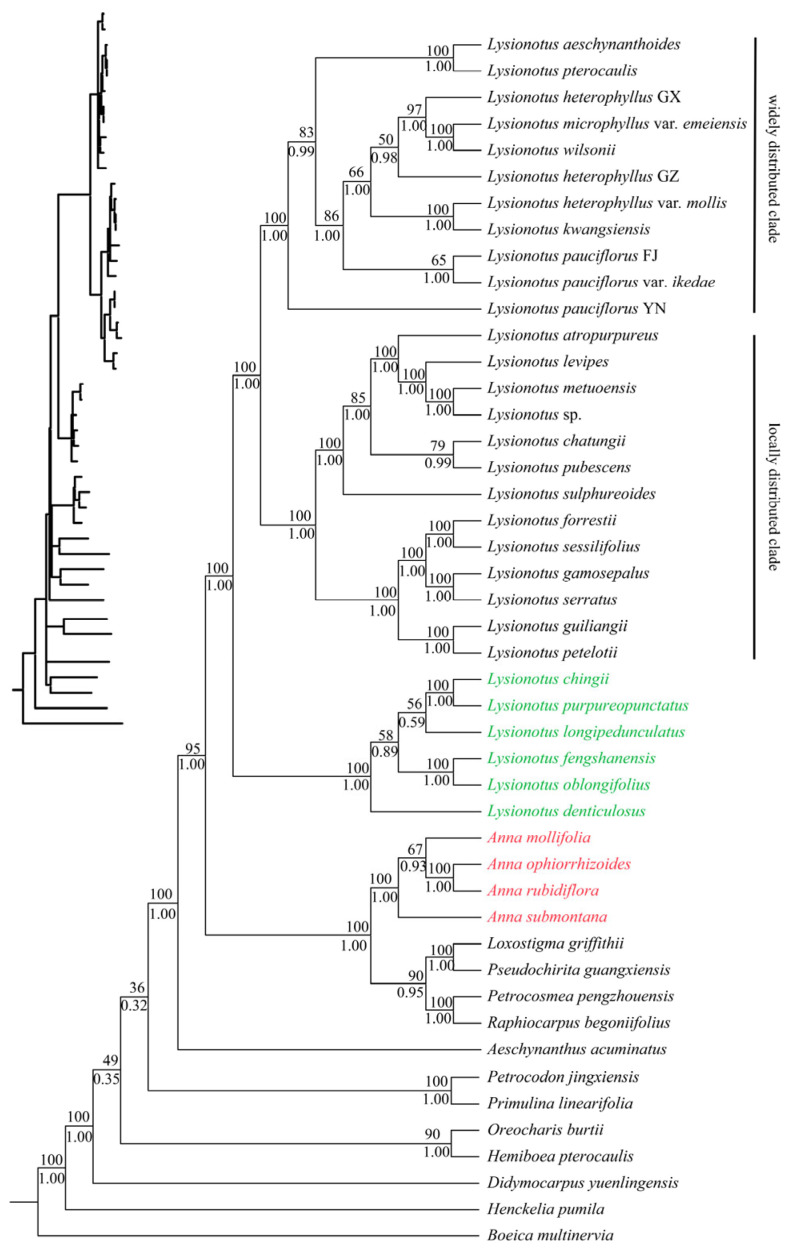
Phylogenetic analysis of ML and BI joint methods based on CDs from 46 chloroplast genomes. BI posterior probability (PP)/ML bootstrap support (BS) values are shown below and above the branch around the corresponding node. The newly obtained chloroplast genomes of *Lysionotus* are shown in green in the figure, and those of *Anna* are shown in red.

The consensus phylogenetic tree (Figure 7) constructed from maximum likelihood (ML) and Bayesian inference (BI) methods based on chloroplast CDs provided a relatively reliable phylogenetic framework. Species representing *Boeica*, *Henckelia*, *Didymocarpus*, *Hemiboea*, *Oreocharis*, *Primulina* and *Petrocodon* were relatively distantly related to the target taxa (*Anna* and *Lysionotus*). Although the genus *Aeschynanthus* belongs to the same tribe as the target group, the phylogenetic relationship between the target taxa and *Aeschynanthus* was relatively distant. All these genera were assigned to the trib. Didymocarpeae, which validated the appropriate selection of outgroups. The target taxa showed relatively close phylogenetic relationship with *Raphiocarpus*, *Petrocosmea* and *Pseudochirita* of the trib. Didymocarpeae. All species of *Anna* formed a well-supported monophyletic group (BS = 100, PP = 1), with clear intrageneric phylogenetic relationships. Meanwhile, the monophyly of *Lysionotus* was confirmed again, as all species of this genus constituted a strongly supported monophyletic group (BS = 100, PP = 1). In the phylogenetic tree, *Anna*, *Raphiocarpus*, *Petrocosmea* and *Pseudochirita* clustered into a well-supported independent clade (BS = 100, PP = 1). This clade was a sister group to *Lysionotus*, sharing the most recent common ancestor (MRCA). The topology structure of this sister group has received strong statistical support (BS = 95, PP = 1). Species of *Lysionotus* were resolved into three clades, which correspond to sects. *Cyathocalyx*, *Didymocarpoides* and *Lysionotus* based on morphological classification, respectively. Sect. *Cyathocalyx* (comprising two species) and sect. *Didymocarpoides* (comprising four species) were clustered into a larger clade with moderate statistical support (BS = 58, PP = 0.89), which was sister to the remaining species of *Lysionotus*. Additionally, sect. *Lysionotus* was divided into two subclades: a widely distributed clade and a locally distributed clade. Eleven samples in the widely distributed subclade formed a monophyletic group, while the locally distributed subclade was further resolved into two clades, consisting of six and seven samples, respectively.

**Figure 8:**
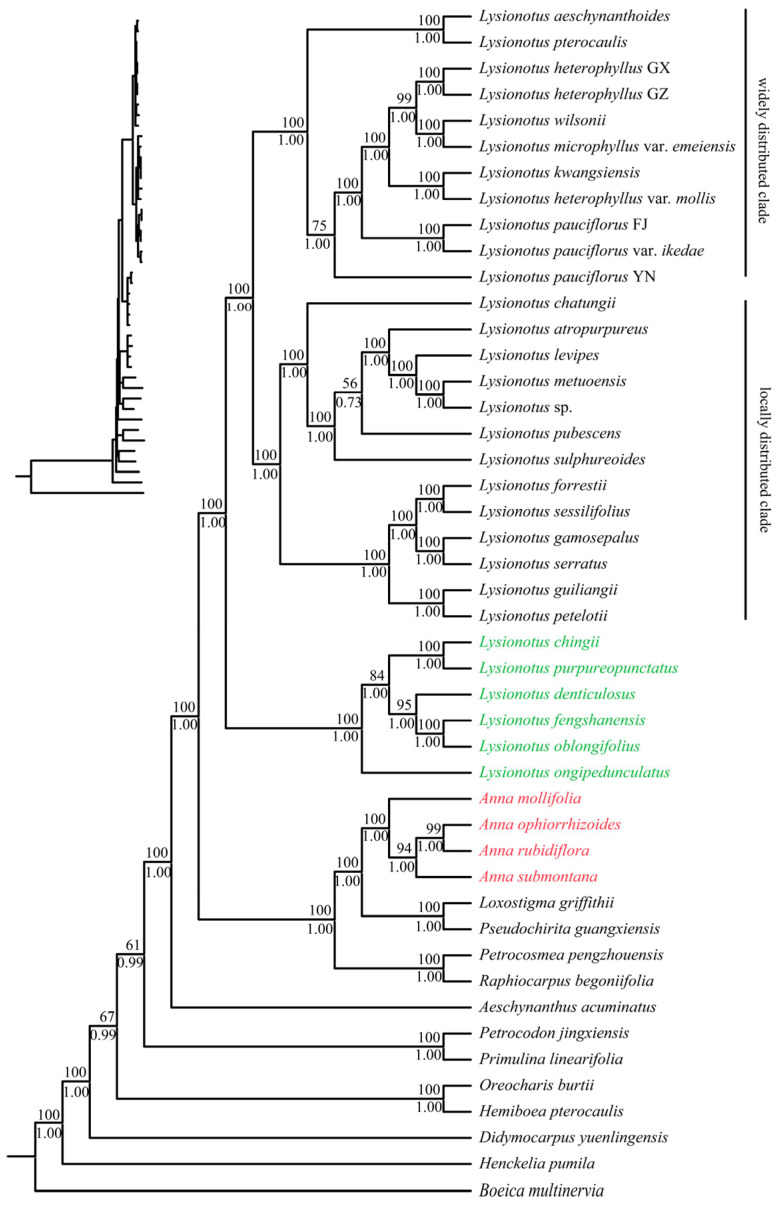
Phylogenetic analysis of ML and BI joint methods based on 46 whole chloroplast genomes. BI posterior probability (PP)/ML bootstrap support (BS) values are shown below and above the branch around the corresponding node. The newly obtained chloroplast genomes of *Lysionotus* are shown in green in the figure, and those of *Anna* are shown in red.

The consensus tree constructed based on the whole chloroplast genome via ML and BI methods clarified the phylogenetic relationships of the target taxa (Figure 8); although its topological structure differs slightly from the consensus tree derived from CDs, the results obtained from the two datasets were not conflicting. Virtually all nodes across the entire phylogenetic tree were strongly supported by high statistical values, indicating a highly resolved and reliable topological structure of the phylogeny. Among the genera belonging to the same trib. as the target taxa, *Aeschynanthus* was distantly related to the target taxa. In contrast, among the genera from another different trib., *Raphiocarpus*, *Petrocosmea* and *Pseudochirita* showed a close phylogenetic relationship with the target taxa. Both *Lysionotus* and *Anna* still formed independent monophyletic groups, respectively. Within *Anna*, *A. rubidiflora* and *A. ophiorrhizoides* showed a relatively close phylogenetic relationship, which was consistent with the topology of the CD tree. In the genus *Lysionotus*, there was a close phylogenetic relationship between *L. purpureopunctatus* and *L. chingii.* In addition, the six species obtained in this study, which represent sects. *Cyathocalyx* and *Didymocarpoides*, were more closely related than to other *Lysionotus* species. The topological relationships exhibited by the species included in sect. Lysionotus were almost consistent with those of the CD datasets.

The topological structure of the neighbor-joining (NJ) tree inferred based on CD dataset deviated markedly from our expectations. We prioritized the congruent topology generated by ML and BI as the core phylogenetic framework, while the NJ tree constructed from the complete plastome dataset was presented only as Supplementary Information (Figure S5). The NJ tree based on the complete plastome also clarified the phylogenetic relationship among outgroups of *Anna* and *Lysionotus*, supporting that both *Anna* and *Lysionotus* formed monophyletically. The four *Anna* species formed a highly supported monophyletic clade (BS = 94). Within *Anna*, *A. rubriflora* and *A. ophiorrhizoides* exhibited the closest phylogenetic relationship (BS = 99), which was consistently validated across three phylogenetic trees. The *Lysionotus* species formed three clades, and the result was validated across all phylogenetic trees.

## 4. Discussion

### 4.1. Evolution of Chloroplast Genome Structure

In this study, we conducted the first comparative analysis of the chloroplast genomes of four *Anna* species and six *Lysionotus* species. The results showed that all chloroplast genomes possess the typical quadripartite structure, with no contraction, expansion or rearrangement of IR regions. This is consistent with the previous research results of *Lysionotus* species [69], confirming the structural stability of chloroplast genomes in both *Lysionotus* and *Anna*.

Among the six *Lysionotus* species, *L. chingii* exhibited the lowest values for overall genomic GC content, GC content of LSC, GC content of the SSC region and GC content of the IR, while simultaneously exhibiting the longest genome length. It constitutes the longest genome with the lowest GC content, suggesting that *L. chingii* prefers AT bases compared to other *Lysionotus* species. For *Anna* species, there was no clear correlation between the lengths of the chloroplast genome and its respective regions (LSC, SSC, IR) and their GC content, with a narrow range of variation. Notably, *L. chingii* (the longest genome and lowest GC content in *Lysionotus*) and *A. ophiorrhizoides* (the longest genome and lowest GC content in *Anna*, with the longest LSC region among congeneric species) share this pattern, which may not be coincidental but rather a result of natural selection acting on these two species.

The low variation in IR region may be attributed to weaker selective pressure on this region, whereas the single-copy regions contain more protein-coding genes that are subject to stronger evolutionary selection pressure [70,71]. Additionally, the high GC content of the IR region is associated with the presence of ribosomal RNA (rRNA) genes, whose secondary structures rely on GC base pairing for stability (GC base pairs form three hydrogen bonds, while AT pairs form only two) [72]. Furthermore, the IR region consists of two inverted repeat sequences; when one repeat undergoes a mutation, the other can serve as a template for repair, thereby further suppressing the accumulation of variations [73].

### 4.2. Sequence Variation and Genetic Diversity

The mVISTA alignment results revealed that the non-coding regions of the chloroplast genomes of *Anna* and *Lysionotus* exhibited significantly higher variation than the coding regions, which is consistent with previous studies [69,74]. Based on the nucleotide diversity (Pi) analysis, *trnH-psbA* (Pi = 0.02767) was the most variable fragment in *Anna*. As a core marker commonly used for plant DNA barcoding and population genetic structure studies [75], the high variability of *trnH-psbA* is usually associated with species adaptive radiation or population expansion events [76]. Fragments including *petB-petD* (Pi = 0.019), *ndhD-psaC* (Pi = 0.01367), *trnL-UAG* (Pi = 0.012), *psaB-psaA* (Pi = 0.012) and *ycf1* (Pi = 0.012) showed moderate genetic diversity, indicating that these fragments maintain a relatively stable mutation rate within *Anna* and can serve as potential molecular markers for population differentiation or phylogenetic analysis. Among them, *ycf1* has been confirmed as one of the most variable coding regions in the chloroplast genomes of angiosperms [77]. The identification of multiple fragments with moderate to high variation in *Anna* based on chloroplast genomes, coupled with an overall medium-to-high level of genetic diversity, suggests that this genus may have experienced population expansion or adaptive differentiation.

In the six *Lysionotus* species, the most variable fragment was also *trnH-psbA*, with moderate genetic diversity (Pi = 0.0196). Compared with the widely distributed and locally distributed Lysionotus species analyzed by Li [69], this value is higher than that of the widely distributed clade (Pi = 0.0152) but lower than that of the locally distributed clade (Pi = 0.02492). This pattern may reflect the relatively narrow distribution range or high gene flow level of the studied *Lysionotus* clade [69]. Fragments such as *petD* (Pi = 0.00973), *clpP* (Pi = 0.0076) and ycf1 (Pi = 0.00827 and 0.00893) exhibited low to moderate genetic diversity (close to the 0.005 threshold). The overall low variation level and weak genetic differentiation among the six *Lysionotus* chloroplast genomes are highly consistent with the genetic characteristics of some endangered species [78]. Combined with the geographical characteristics of the China–Vietnam border, the geographical isolation caused by the complex karst terrain in this region has restricted gene flow among species, which further promoted the genetic differentiation of hypervariable regions in the two genera [79].

Results of SSRs and LRSs revealed the evolutionary characteristics of repetitive sequences in the two genera: SSRs in both genera were dominated by mononucleotide repeats (predominantly A/T), consistent with the universal rule that plant chloroplast SSRs are rich in AT [69], and interspecific differences in such repetitive sequences can serve as efficient markers for species identification. Meanwhile, distinct generic-specific patterns of SSRs were observed between the two genera.

Four types of LRSs were identified in *Anna* species: palindromic repeats, forward repeats, reverse repeats and complementary repeats. Among the detected repeats, palindromic and forward repeats exhibited the wider ranges (16–25 and 12–20, respectively), with their maximum in *A. ophiorrhizoides*, while reverse repeats were rare (1–3), with only one complementary repeat being identified in *A. rubidiflora.* Only three types of LRSs were found in six *Lysionotus* species and no complementary repeats were detected. There were 16 consistent palindromic repeats, 13 forward repeats (with the exception of 14 for *L. longipedunculatus*), and one reverse repeat exclusively identified in *L. longipedunculatus*. In summary, the types and numbers of long repeat sequences were highly conserved among the studied *Lysionotus* species from the Sino-Vietnamese border region. Palindromic repeats (P) and forward repeats (F) play key roles in maintaining chloroplast genome stability, regulating gene expression under abiotic stresses and promoting adaptive evolution via homologous recombination. Reverse repeats (R) have limited regulatory functions and complementary repeats (C) are non-functional and rare. The predominance of P and F over R and C in *Anna* and *Lysionotus* species is a conserved pattern, reflecting a balance between genome stability and mutability for environmental adaptation, which has been verified in multiple angiosperm genera [80,81].

### 4.3. Phylogenetic Relationships Based on Chloroplast Genomes

ML and BI analyses based on complete chloroplast genomes and CD datasets consistently confirmed that *Anna* and *Lysionotus* each form highly supported monophyletic clades (BS = 100, PP = 1) and are sister genera, supporting the treatment by Moller [20], further validating their stable phylogenetic positions in trib. Trichosporeae. In the phylogenetic tree, *Petrocosmea*, *Raphiocarpus* and *Pseudochirita* of trib. Trichosporeae are nested within the sister clade containing *Anna*, suggesting gene flow among these genera. All *Lysionotus* species formed in a strongly supported monophyletic group, and the close relationships were confirmed. The phylogenetic clades of *Lysionotus* are highly consistent with morphological classification: the six studied species correspond to *sect. Cyathocalyx* and *sect. Didymocarpoides*, which cluster into a larger clade sister to *sect. Lysionotus*. The topological structures of *sect. Cyathocalyx* and *sect. Didymocarpoides* differ slightly from the relationships obtained based on the transcriptome [12], which might be because of nuclear–cytoplasmic conflicts, with the core phylogenetic framework derived from the chloroplast genome and transcriptome consistently. Furthermore, the phylogenetic relationships of *Lysionotus* may be associated with geographical distribution, where widely distributed and locally distributed clades cluster separately, supporting that topographic heterogeneity along the China–Vietnam border and Miocene climate fluctuations jointly drove the species diversification of *Lysionotus* [12].

Significant topological differences between the NJ phylogenetic tree and the consensus tree based on CDs mainly result from the mismatch between algorithmic logic and the evolutionary characteristics of chloroplast genomes. As a distance-based approach, the NJ method is built upon the molecular clock hypothesis of constant sequence evolution, and its application requires simplifying both site-specific evolutionary heterogeneity and base substitution model complexity [82,83]. Furthermore, the slight topological differences between the consensus tree based on the CDs dataset and complete chloroplast genome may be caused by the number of phylogenetic signal sites, and incomplete lineage sorting (ILS) may also contribute to the topological incongruence [84,85,86].

### 4.4. Relatively Independent Adaptive Evolution in a Sympatric Distribution Context

The species-specific mutations observed, along with the monophyletic groups with high support, provide evidence for independent evolution. Although the taxa of these species are geographically coexisting, they exhibit different evolutionary characteristics, which is likely driven by their specific adaptive patterns in microhabitats. Special habitats exert a strong environmental filtering effect, and only individuals with specific adaptive genetic backgrounds survived and reproduced [87].

Independent evolution is also reflected in the divergence of their morphological traits. As epiphytic/lithophytic taxa in Gesneriaceae, both genera retain basic morphological adaptations to rocky habitats (e.g., succulent stems and leathery leaves) [4,14]. However, Lysionotus exhibits climbing or creeping growth habits, which may facilitate its climbing and long-distance seed dispersal [14,69]; it inhabits habitats at elevations of 300–2000 m, with a flowering period from July to October. In contrast, Anna grows erect, occupies habitats at 800–1700 m, and flowers from June to October [14]. The non-complete overlap in their habitat elevations and phenology may have promoted niche differentiation between the two genera in sympatric environments, enabling them to maintain independent evolution within similar habitats [87,88].

### 4.5. Novel Contributions, Limitations and Future Perspectives

This study performed comparative chloroplast genome and phylogenetic analyses of *Anna* and *Lysionotus* along the Sino-Vietnamese border, revealing their structural characteristics, sequence variation and phylogenetic relationships. It provides cases and insights for closely related genera of endemic plants and their sympatric distribution, and how they evolved relatively independently to adapt to the special karst habitat. But the study only focused on genomic evolutionary patterns, failing to associate adaptive evolution with specific habitat indicators (e.g., soil properties) and lacking supportive physiological data, limiting clarification of links between genomic variation, ecological adaptation and phenotypic functions.

Moreover, common bottlenecks in organelle genomics restrict chloroplast genome data mining, including incomplete annotation of unknown-function ORFs, unclear dynamic population variation and 3D conformation of chloroplast genomes across tissues/developmental stages, unelucidated associations between the replication–recombination–repair mechanism and genomic variations, lack of standardized systems for intracellular horizontal gene transfer, and insufficient understanding of nuclear–chloroplast coordinated gene expression [29].

## 5. Conclusions

This is the first report comparing the complete chloroplast genome between all *Anna* genus and related *Lysionotus* species distributed along the border area between China and Vietnam. In this study, 10 chloroplast genomes had typical quadripartite structure, without significant contraction or expansion. The number of CDs, rRNA and tRNA varied among different species, whereas they were relatively conserved between the two genera. Based on the distinctly high Pi values observed, *psaB-psaA*, *trnL-UAG* and *ndhD-psaC* were identified as potential molecular markers for *Anna*. In contrast, *clpP* and *ycf1* were proposed as effective molecular markers for *Lysionotus*. Additionally, the types of SSRs and LRSs exhibited higher conservation in *Lysionotus* compared with *Anna*. Furthermore, the codon usage preferences of the two genera showed convergent evolutionary trends, and natural selection played a dominant role in shaping these preference patterns. Notably, ycf1 and *atpH* were confirmed as significantly positively selected genes, which may contribute to their adaptation to the unique Sino-Vietnamese karst habitats. Phylogenetic analyses using multiple approaches (ML, BI and NJ) consistently verified that each genus forms a well-supported monophyletic clade and shares a common ancestor, indicating a close phylogenetic relationship between *Anna* and *Lysionotus*.

## Figures and Tables

**Table 1:** Taxa information of plastomes used in this study.

Species Name	Voucher	Herbarium	Locality	Collector
*Anna mollifolia*	14748	IBK	Napo County, Guangxi Zhuang Autonomous Region, China	Weibin Xu et al.
*Anna ophiorrhizoides*	14205	IBK	Leshan City, Sichuan Province, China	Weibin Xu
*Anna rubidiflora*	21062501	HGAS	Guizhou Botanical Garden, Guiyang City, China	Shenghu Tang
*Anna submontana*	13945	HN	HaGiang, Vietnam	Khang Sinh Nguyen et al.
*Lysionotus denticulosus*	091706	IBK	Huanjiang County, Guangxi Zhuang Autonomous Region, China	Weibin Xu
*Lysionotus longipedunculatus*	0027	IBK	Jingxi City, Guangxi Zhuang Autonomous Region, China	Zhirong Liu
*Lysionotus oblongifolius*	14900	IBK	Jingxi City, Guangxi Zhuang Autonomous Region, China	Weibin Xu et al.
*Lysionotus chingii*	14747	IBK	Napo County, Guangxi Zhuang Autonomous Region, China	Weibin Xu et al.
*Lysionotus fengshanensis*	L1277	IBK	Fengshan County, Guangxi Zhuang Autonomous Region, China	Yan Liu et al.
*Lysionotus purpureopunctatus*	14229	IBK	Guilin Botanical Garden, Guilin City, China	Weibin Xu

**Table 2:** Basic characteristics of *Anna* and *Lysionotus* species chloroplast genomes (LSC, large single-copy region; SSC, small single-copy region; IR, inverted repeat regions; GC%, guanine–cytosine percentage; length expressed in base pairs, bp).

Species Name	Size (bp)	LSC (bp)	SSC (bp)	IR (bp)	GC Content				CDs	tRNA Genes	rRNA Genes
					Total	LSC	SSC	IR			
*Anna mollifolia*	154,012	84,959	18,329	25,362	37.49	35.42	31.17	43.23	88	38	8
*Anna ophiorrhizoides*	154,246	85,104	18,350	25,396	37.44	35.37	31.08	43.20	85	36	8
*Anna rubidiflora*	154,011	85,002	18,283	25,363	37.47	35.39	31.19	43.22	87	36	8
*Anna submontana*	154,088	85,010	18,360	25,359	37.44	35.37	31.04	43.23	88	38	8
*Lysionotus denticulosus*	154,196	85,058	18,250	25,444	37.53	35.49	31.26	43.20	87	36	8
*Lysionotus longipedunculatus*	154,198	85,066	18,220	25,456	37.58	35.54	31.38	43.20	87	35	8
*Lysionotus oblongifolius*	154,184	85,047	18,235	25,451	37.55	35.52	31.25	43.19	87	35	8
*Lysionotus chingii*	154,278	85,144	18,210	25,462	37.48	35.42	31.18	43.17	87	35	8
*Lysionotus fengshanensis*	154,256	85,126	18,226	25,452	37.57	35.54	31.36	43.21	87	35	8
*Lysionotus purpureopunctatus*	154,276	85,113	18,199	25,467	37.50	35.44	31.22	43.19	87	35	8

**Table 3:** GC content statistics across distinct codon positions in organelle genome coding sequences.

Species Name	GC1	GC2	GC3	GC All Everage	ENC	GC3s
*Anna mollifolia*	46.59	39.50	27.80	37.96	45.15	27.85
*Anna ophiorrhizoides*	46.40	39.48	27.95	37.94	45.65	28.00
*Anna rubidiflora*	46.45	39.58	27.94	37.99	45.38	27.98
*Anna submontana*	46.57	39.55	27.79	37.97	45.12	27.85
*Lysionotus chingii*	46.54	39.54	27.94	38.01	45.24	27.99
*Lysionotus denticulosus*	46.61	39.58	27.91	38.03	45.06	27.97
*Lysionotus fengshanensis*	46.60	39.57	27.97	38.05	45.33	28.02
*Lysionotus longipedunculatus*	46.59	39.56	28.03	38.06	45.32	28.08
*Lysionotus oblongifolius*	46.58	39.55	27.92	38.02	45.30	27.96
*Lysionotus purpureopunctatus*	46.57	39.54	27.92	38.01	45.28	27.98

## Data Availability

The data generated in this study are available at Genbank (accession numbers: PQ468964–PQ468966, PQ468979–PQ468982, PQ468984–PQ468985, PQ468995).

## Supplementary Materials

The following supporting information can be downloaded at: https://www.mdpi.com/article/10.3390/biology15040352/s1. Figure S1: Comparative alignment of 10 chloroplast genomes with *Lysionotus longipedunculatus* as a reference; Figure S2: ENC plot analysis of 10 chloroplast genomes; Figure S3: PR2 plot analysis of 10 chloroplast genomes; Figure S4: Neutrality plot analysis of 10 chloroplast genomes; Figure S5: NJ phylogenetic tree based on the complete chloroplast genome datasets. Table S1: Public data information for reconstructing phylogenetic relationships in this study; Table S2: Potential positive selection test based on the branch-site model; Table S3: Statistics of genes within *Anna*; Table S4: Statistics of genes within *Lysionotus.*

## Abbreviations

The following abbreviations are used in this manuscript:

LSC	Large single-copy
SSC	Small single-copy
IR	Inverted repeat
IGS	Intergenic spacer
Pi	Nucleotide diversity
SSRs	Simple sequence repeats
LSRs	Large sequence repeats
CDs	Coding sequences
RSCU	Relative synonymous codon usage
dN	Nonsynonymous substitution rate
dS	Synonymous substitution rate
ω	Nonsynonymous to synonymous substitution rate ratios
ML	Maximum likelihood
BI	Bayesian inference
NJ	Neighbor-joining
BS	Bootstrap support
PP	Posterior probabilities
