# Supplementary material for: Comparative Chloroplast Genome and Phylogenetic Analyses of Anna and Lysionotus (Gesneriaceae) Along the Sino-Vietnamese Border
Source: Biology (Basel). 2026 Feb 18;15(4):352. doi: 10.3390/biology15040352 (PMC12938752; doi:10.3390/biology15040352)
Supplement: Supplementary file 1 [file biology-15-00352-s001.zip › Figure S1_mVISTA.pdf]

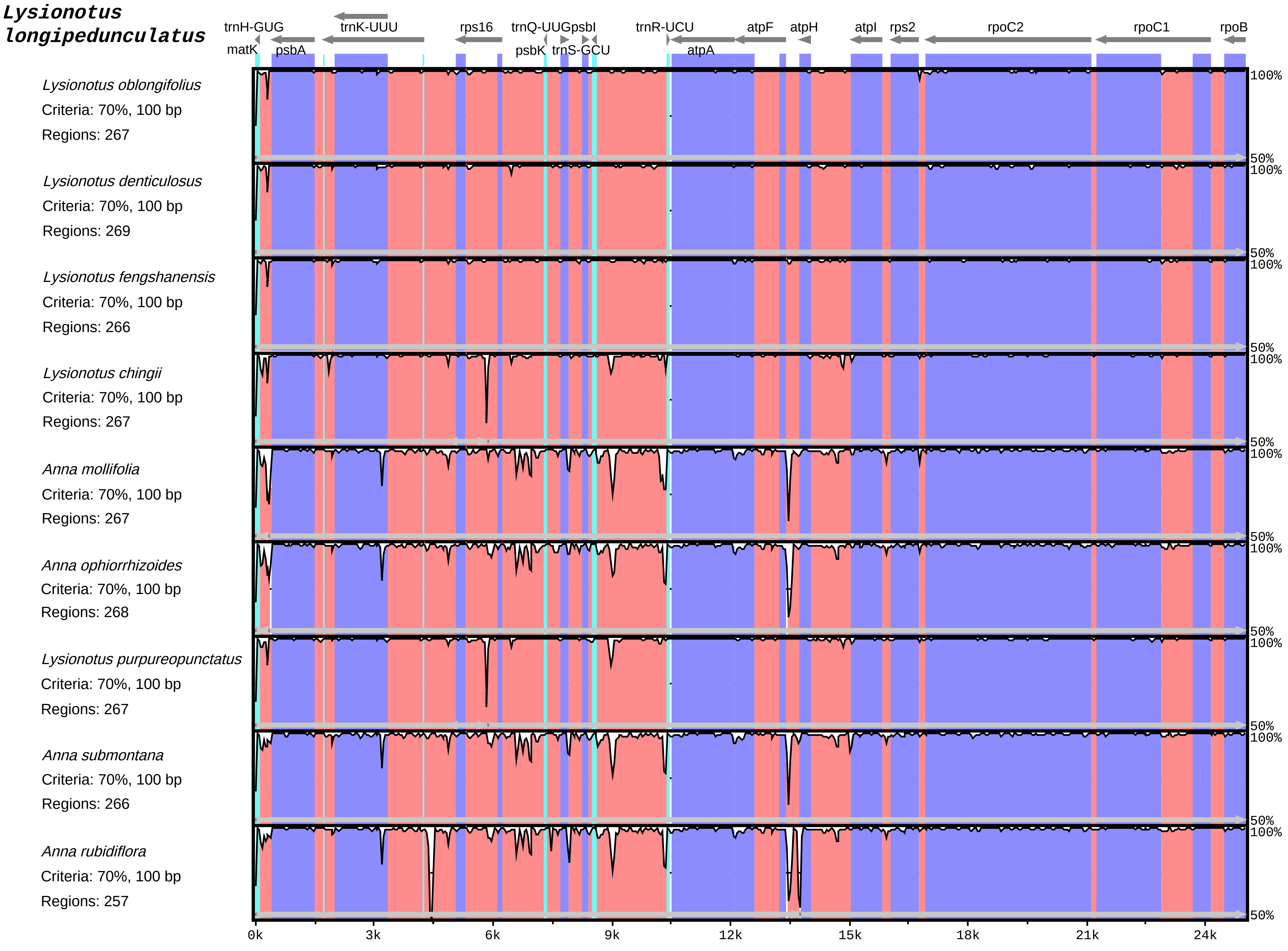

Lysionotus longipedunculatus
trnH-GUG
matK
psbA
trnK-UUU
rps16
trnQ-UUG
psbK
psbI
trnS-GCU
trnR-UCU
atpA
atpF
atpH
atpI
rps2
rpoC2
rpoC1
rpoB
Lysionotus oblongifolius
Criteria: 70%, 100 bp
Regions: 267
Lysionotus denticulosus
Criteria: 70%, 100 bp
Regions: 269
Lysionotus fengshanensis
Criteria: 70%, 100 bp
Regions: 266
Lysionotus chingii
Criteria: 70%, 100 bp
Regions: 267
Anna mollifolia
Criteria: 70%, 100 bp
Regions: 267
Anna ophiorrhizoides
Criteria: 70%, 100 bp
Regions: 268
Lysionotus purpureopunctatus
Criteria: 70%, 100 bp
Regions: 267
Anna submontana
Criteria: 70%, 100 bp
Regions: 266
Anna rubidiflora
Criteria: 70%, 100 bp
Regions: 257
100%
50%
0k
3k
6k
9k
12k
15k
18k
21k
24k
X-axis: L.longipedunculatus
Resolution: 39
Window size: 100 bp

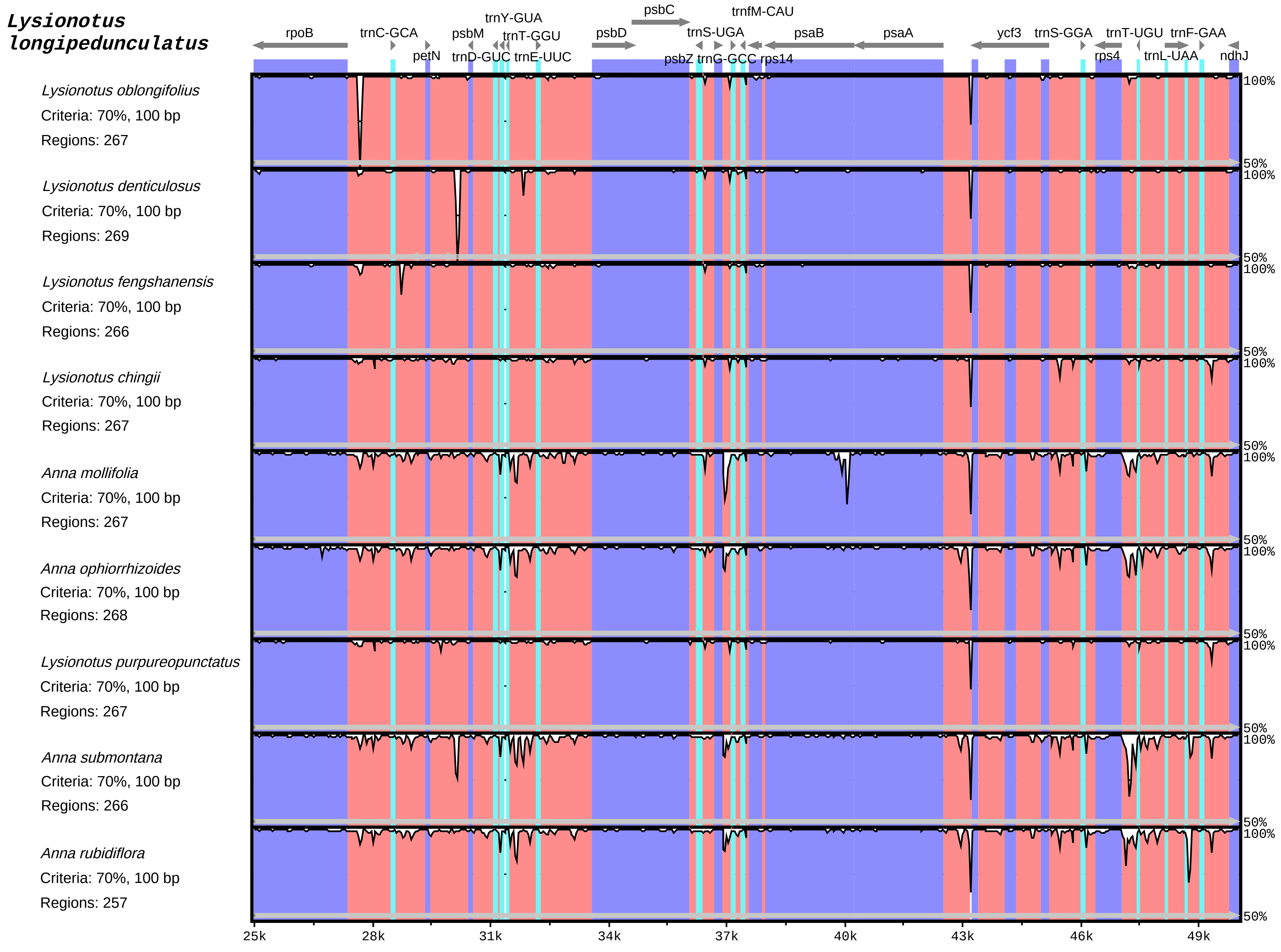
Lysionotus longipedunculatus
rpoB
trnC-GCA
petN
psbM
trnY-GUA
trnD-GUC
trnT-GGU
trnE-UUC
psbD
psbC
trnS-UGA
psbZ
trnG-GCC
trnfM-CAU
rps14
psaB
psaA
ycf3
trnS-GGA
rps4
trnT-UGU
trnL-UAA
trnF-GAA
ndhJ
Lysionotus oblongifolius
Criteria: 70%, 100 bp
Regions: 267
Lysionotus denticulosus
Criteria: 70%, 100 bp
Regions: 269
Lysionotus fengshanensis
Criteria: 70%, 100 bp
Regions: 266
Lysionotus chingii
Criteria: 70%, 100 bp
Regions: 267
Anna mollifolia
Criteria: 70%, 100 bp
Regions: 267
Anna ophiorrhizoides
Criteria: 70%, 100 bp
Regions: 268
Lysionotus purpureopunctatus
Criteria: 70%, 100 bp
Regions: 267
Anna submontana
Criteria: 70%, 100 bp
Regions: 266
Anna rubidiflora
Criteria: 70%, 100 bp
Regions: 257
100%
50%
25k
28k
31k
34k
37k
40k
43k
46k
49k

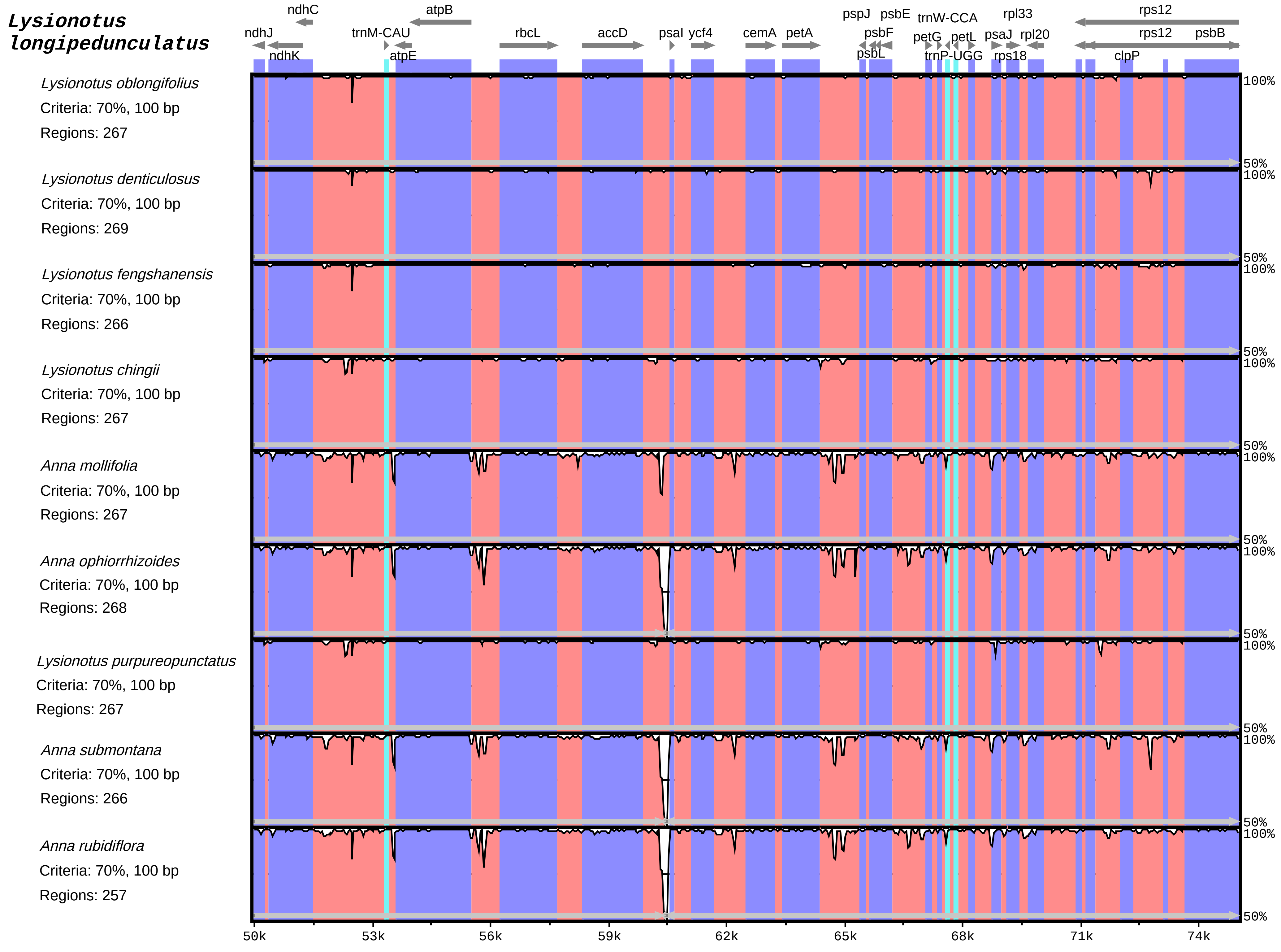

Lysionotus longipedunculatus
ndhC
atpB
ndhJ
ndhK
trnM-CAU
atpE
rbcL
accD
psaI
ycf4
cemA
petA
pspJ
psbE
psbF
psbL
trnW-CCA
petG
petL
trnP-UGG
psaJ
rpl33
rps18
rpl20
rps12
rps12
psbB
clpP
Lysionotus oblongifolius
Criteria: 70%, 100 bp
Regions: 267
Lysionotus denticulosus
Criteria: 70%, 100 bp
Regions: 269
Lysionotus fengshanensis
Criteria: 70%, 100 bp
Regions: 266
Lysionotus chingii
Criteria: 70%, 100 bp
Regions: 267
Anna mollifolia
Criteria: 70%, 100 bp
Regions: 267
Anna ophiorrhizoides
Criteria: 70%, 100 bp
Regions: 268
Lysionotus purpureopunctatus
Criteria: 70%, 100 bp
Regions: 267
Anna submontana
Criteria: 70%, 100 bp
Regions: 266
Anna rubidiflora
Criteria: 70%, 100 bp
Regions: 257
100%
50%
50k
53k
56k
59k
62k
65k
68k
71k
74k

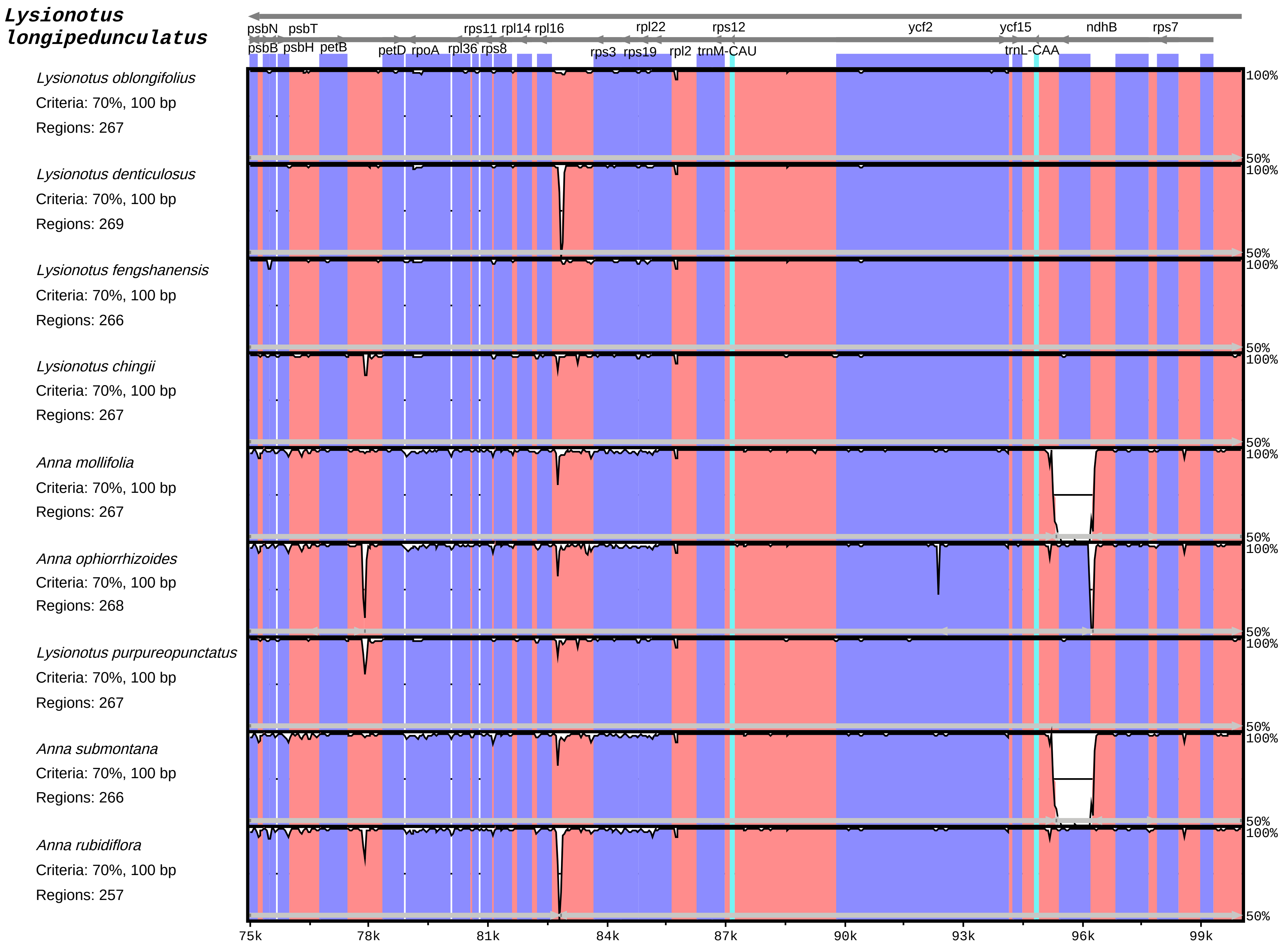
Lysionotus longipedunculatus
psbN
psbT
rps11
rpl14
rpl16
rpl22
rps12
ycf2
ycf15
ndhB
rps7
psbB
psbH
petB
petD
rpoA
rpl36
rps8
rps3
rps19
rpl2
trnM-CAU
trnL-CAA
Lysionotus oblongifolius
Criteria: 70%, 100 bp
Regions: 267
Lysionotus denticulosus
Criteria: 70%, 100 bp
Regions: 269
Lysionotus fengshanensis
Criteria: 70%, 100 bp
Regions: 266
Lysionotus chingii
Criteria: 70%, 100 bp
Regions: 267
Anna mollifolia
Criteria: 70%, 100 bp
Regions: 267
Anna ophiorrhizoides
Criteria: 70%, 100 bp
Regions: 268
Lysionotus purpureopunctatus
Criteria: 70%, 100 bp
Regions: 267
Anna submontana
Criteria: 70%, 100 bp
Regions: 266
Anna rubidiflora
Criteria: 70%, 100 bp
Regions: 257
100%
50%
75k
78k
81k
84k
87k
90k
93k
96k
99k

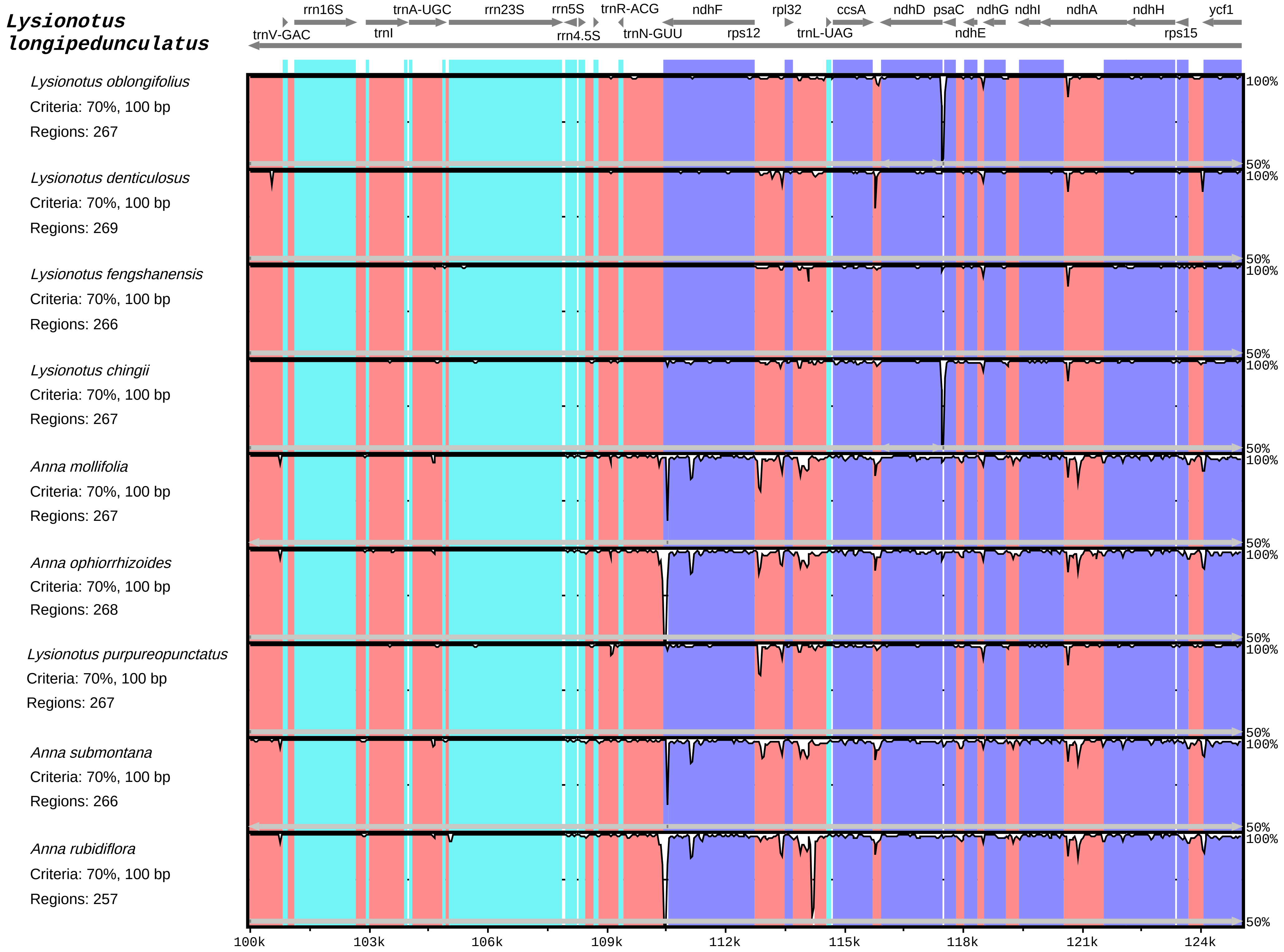

Lysionotus longipedunculatus
rrn16S
trnA-UGC
rrn23S
rrn5S
trnR-ACG
ndhF
rpl32
ccsA
ndhD
psaC
ndhG
ndhI
ndhA
ndhH
ycf1
trnV-GAC
trnI
rrn4.5S
trnN-GUU
rps12
trnL-UAG
ndhE
rps15
Lysionotus oblongifolius
Criteria: 70%, 100 bp
Regions: 267
Lysionotus denticulosus
Criteria: 70%, 100 bp
Regions: 269
Lysionotus fengshanensis
Criteria: 70%, 100 bp
Regions: 266
Lysionotus chingii
Criteria: 70%, 100 bp
Regions: 267
Anna mollifolia
Criteria: 70%, 100 bp
Regions: 267
Anna ophiorrhizoides
Criteria: 70%, 100 bp
Regions: 268
Lysionotus purpureopunctatus
Criteria: 70%, 100 bp
Regions: 267
Anna submontana
Criteria: 70%, 100 bp
Regions: 266
Anna rubidiflora
Criteria: 70%, 100 bp
Regions: 257
100%
50%
100k
103k
106k
109k
112k
115k
118k
121k
124k

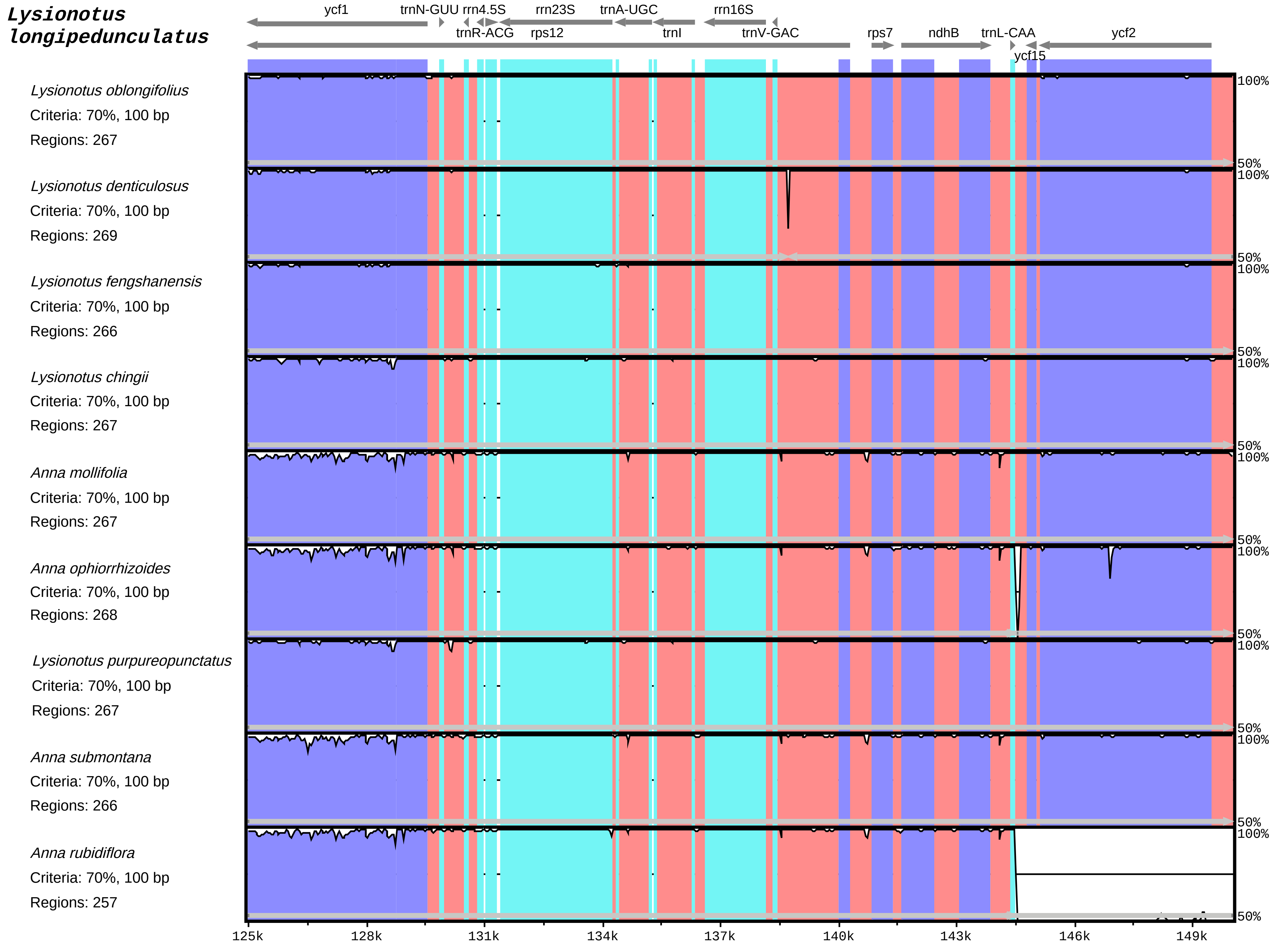
Lysionotus longipedunculatus
ycf1
trnN-GUU
rrn4.5S
rrn23S
trnA-UGC
rrn16S
trnR-ACG
rps12
trnI
trnV-GAC
rps7
ndhB
trnL-CAA
ycf2
ycf15
Lysionotus oblongifolius
Criteria: 70%, 100 bp
Regions: 267
Lysionotus denticulosus
Criteria: 70%, 100 bp
Regions: 269
Lysionotus fengshanensis
Criteria: 70%, 100 bp
Regions: 266
Lysionotus chingii
Criteria: 70%, 100 bp
Regions: 267
Anna mollifolia
Criteria: 70%, 100 bp
Regions: 267
Anna ophiorrhizoides
Criteria: 70%, 100 bp
Regions: 268
Lysionotus purpureopunctatus
Criteria: 70%, 100 bp
Regions: 267
Anna submontana
Criteria: 70%, 100 bp
Regions: 266
Anna rubidiflora
Criteria: 70%, 100 bp
Regions: 257
100%
50%
125k
128k
131k
134k
137k
140k
143k
146k
149k

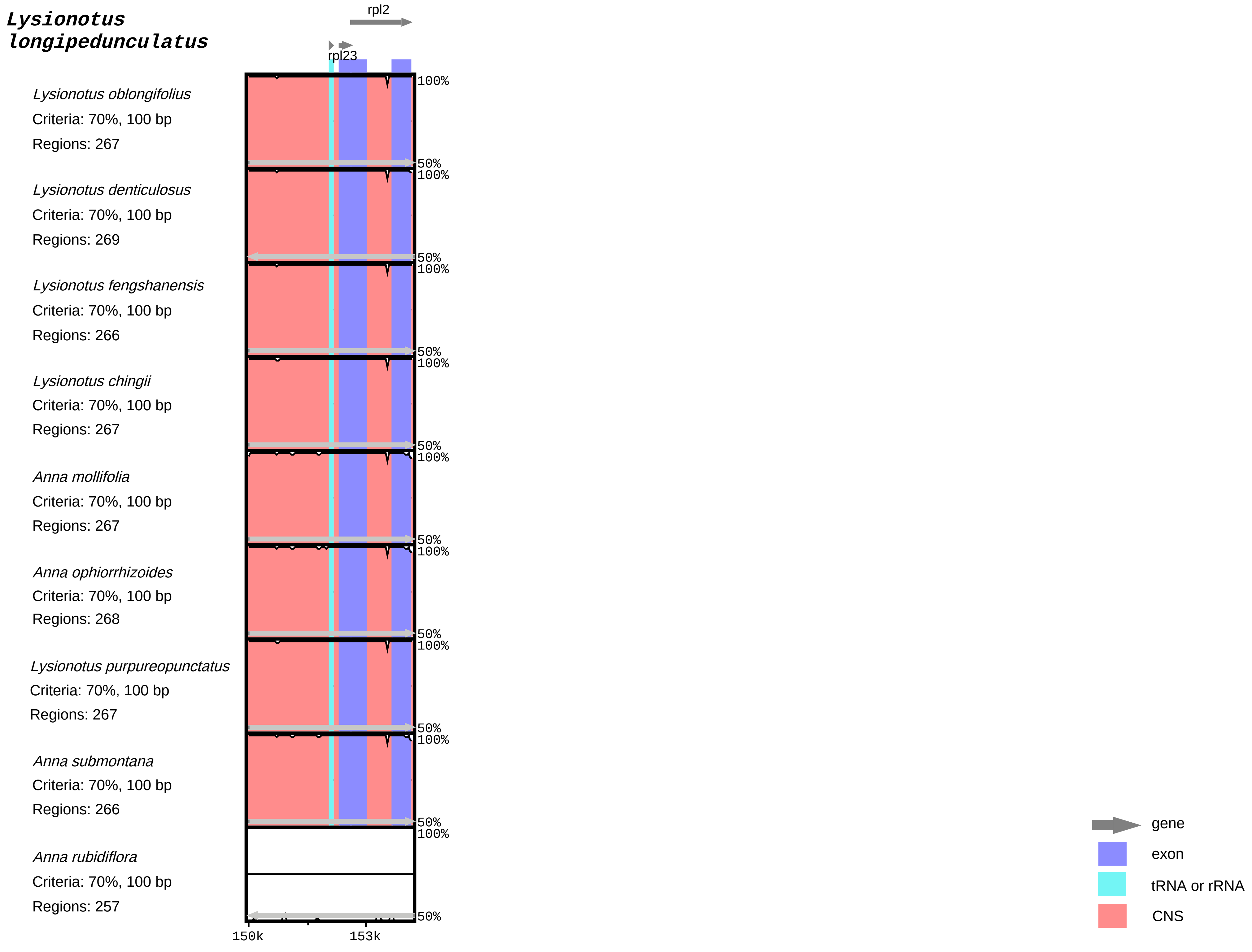
Lysionotus longipedunculatus
rpl2
rpl23
Lysionotus oblongifolius
Criteria: 70%, 100 bp
Regions: 267
Lysionotus denticulosus
Criteria: 70%, 100 bp
Regions: 269
Lysionotus fengshanensis
Criteria: 70%, 100 bp
Regions: 266
Lysionotus chingii
Criteria: 70%, 100 bp
Regions: 267
Anna mollifolia
Criteria: 70%, 100 bp
Regions: 267
Anna ophiorrhizoides
Criteria: 70%, 100 bp
Regions: 268
Lysionotus purpureopunctatus
Criteria: 70%, 100 bp
Regions: 267
Anna submontana
Criteria: 70%, 100 bp
Regions: 266
Anna rubidiflora
Criteria: 70%, 100 bp
Regions: 257
100%
50%
150k
153k
gene
exon
tRNA or rRNA
CNS
