# Supplementary material for: Comparative Chloroplast Genome and Phylogenetic Analyses of Anna and Lysionotus (Gesneriaceae) Along the Sino-Vietnamese Border
Source: Biology (Basel). 2026 Feb 18;15(4):352. doi: 10.3390/biology15040352 (PMC12938752; doi:10.3390/biology15040352)
Supplement: Supplementary file 1 [file biology-15-00352-s001.zip › Table S1.The public data information for reconstructing phylogenetic relationships in this study.pdf]

Table S1. The public data information for reconstructing phylogenetic relationships in this study

**Species**	**Genbank accession**
Aeschynanthus acuminatus	PX741072.1
Boeica multinervia	NC_070432.1
Didymocarpus yuenlingensis	OR666670.1
Hemiboea pterocaulis	NC_082106.1
Henckelia pumila	NC_065361.1
Loxostigma griffithii	NC_082153.1
Oreocharis burtii	NC_086552.1
Petrocodon jingxiensis	NC_044477.1
Petrocosmea pengzhouensis	PV750053.1
Primulina linearifolia	NC_036414.1
Pseudochirita guangxiensis	NC_082154.1
Raphiocarpus begoniifolia	NC_082152.1
Lysionotus pauciflorus	PQ433130.1
Lysionotus pauciflorus	PQ468987.1
Lysionotus pauciflorus var. *ikedae*	PQ468992.1
Lysionotus aeschynanthoides	PQ468968.1
Lysionotus pterocaulis	PQ468977.1
Lysionotus heterophyllus	PQ468988.1
Lysionotus wilsonii	PQ468969.1
Lysionotus microphyllus var. *omeiensis*	PQ468994.1
Lysionotus heterophyllus	PQ468967.1
Lysionotus kwangsiensis	PQ468975.1
Lysionotus heterophyllus var. *mollis*	PQ468990.1
Lysionotus gamosepalus	PQ468978.1
Lysionotus pubescens	PQ468973.1
Lysionotus atropurpureus	PQ468986.1
Lysionotus sessilifolius	PQ468971.1
Lysionotus forrestii	PQ468993.1
Lysionotus levipes	PQ468989.1
Lysionotus chatungii	PQ468991.1
Lysionotus guiliangii ined.	PQ468983.1
Lysionotus sp.	PQ468996.1
Lysionotus petelotii	PQ468970.1
Lysionotus sulphureoides	PQ468972.1
Lysionotus serratus	PQ468976.1
Lysionotus metuoensis	PQ468974.1
