# Supplementary figures and images for: Comparative Chloroplast Genome and Phylogenetic Analyses of Anna and Lysionotus (Gesneriaceae) Along the Sino-Vietnamese Border
Source: Biology (Basel). 2026 Feb 18;15(4):352. doi: 10.3390/biology15040352 (PMC12938752; doi:10.3390/biology15040352)

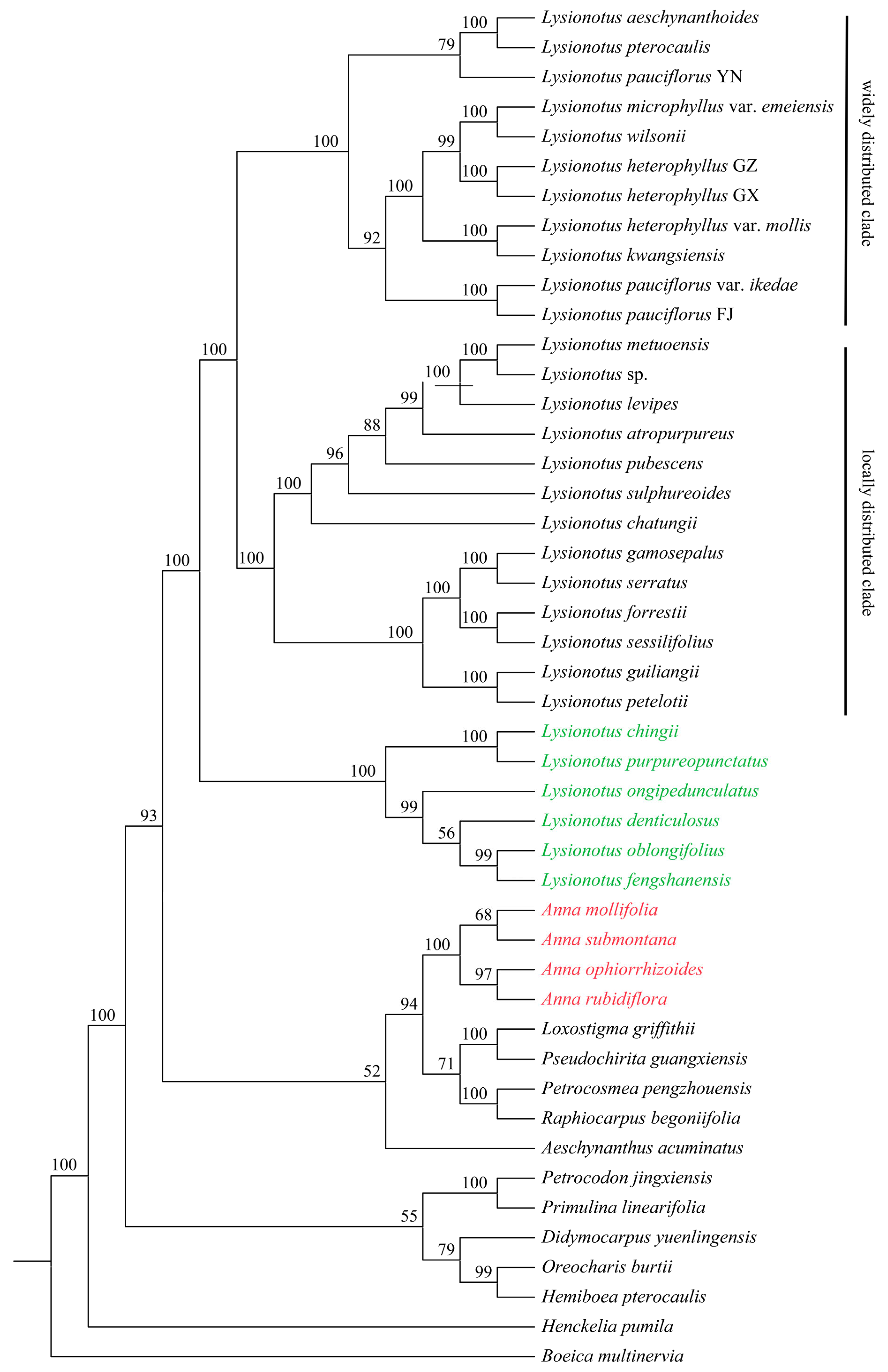

Supplement: Supplementary file 1 [file biology-15-00352-s001.zip › Figure S5_46_fasta_NJ_tree.tif]
